# NMR structure of a G-quadruplex formed by four d(G_4_C_2_) repeats: insights into structural polymorphism

**DOI:** 10.1093/nar/gky886

**Published:** 2018-10-02

**Authors:** Jasna Brčić, Janez Plavec

**Affiliations:** 1 Slovenian NMR Center, National Institute of Chemistry, Ljubljana SI-1000, Slovenia; 2 Faculty of Chemistry and Chemical Technology, University of Ljubljana, Ljubljana SI-1000, Slovenia; 3 EN-FIST Center of Excellence, Ljubljana SI-1000, Slovenia

## Abstract

Most frequent genetic cause of amyotrophic lateral sclerosis (ALS) and frontotemporal dementia (FTD), is a largely increased number of d(G_4_C_2_)n•(G_2_C_4_)n repeats located in the non-coding region of *C9orf72* gene. Non-canonical structures, including G-quadruplexes, formed within expanded repeats have been proposed to drive repeat expansion and pathogenesis of ALS and FTD. Oligonucleotide d[(G_4_C_2_)_3_G_4_], which represents the shortest oligonucleotide model of d(G_4_C_2_) repeats with the ability to form a unimolecular G-quadruplex, forms two major G-quadruplex structures in addition to several minor species which coexist in solution with K^+^ ions. Herein, we used solution-state NMR to determine the high-resolution structure of one of the major G-quadruplex species adopted by d[(G_4_C_2_)_3_G_4_]. Structural characterization of the G-quadruplex named AQU was facilitated by a single substitution of dG with 8Br-dG at position 21 and revealed an antiparallel fold composed of four G-quartets and three lateral C–C loops. The G-quadruplex exhibits high thermal stability and is favored kinetically and under slightly acidic conditions. An unusual structural element distinct from a C-quartet is observed in the structure. Two C•C base pairs are stacked on the nearby G-quartet and are involved in a dynamic equilibrium between symmetric N3-amino and carbonyl-amino geometries and protonated C^+^•C state.

## INTRODUCTION

An increase in the number of d(G_4_C_2_)•(G_2_C_4_) nucleotide repeats (repeat expansion) in the non-coding region of *C9orf72* gene has been identified as the most common genetic cause of two devastating neurological disorders, amyotrophic lateral sclerosis (ALS) and frontotemporal dementia (FTD) (1,2). Between 2 and 23 repeats of d(G_4_G_2_) are typical in a healthy population, whereas people with ALS and FTD can carry >1000 of d(G_4_G_2_) repeats (1). The ability of a repetitive DNA sequence to adopt a non-canonical secondary structure during replication, transcription or DNA repair is considered crucial in the proposed molecular mechanisms, which are responsible for repeat expansion (3–6). DNA and RNA oligonucleotides containing (G_4_C_2_) and (G_2_C_4_) repeats have been shown to fold into a G-quadruplex and other non-canonical structures (7–10). Moreover, the d(G_4_C_2_)•(G_2_C_4_) repeats are unstable during transcription and replication *in vitro* (11,12). While the mechanism through which this expanded repeat causes neurodegeneration remains unknown, a mechanism has been proposed which includes a non-canonical structure formation on DNA and RNA level. The non-canonical structures including G-quadruplexes and RNA•DNA hybrids form within d(G_4_C_2_)n•(G_2_C_4_)*_n_* repeats and stall RNA polymerase. This leads to a lack of completely transcribed *C9orf72* mRNA as well as production of abortive RNA transcripts, which contain (G_4_C_2_) and (G_2_C_4_) repeats. Decreased level of C9ORF72 protein (2,13,14), which is involved in clearance of stress granules (15), has been shown recently to contribute to neurodegeneration (16). Further pathology arises from a gain-of-function mechanism imparted by RNA transcripts of both sense d(G_4_G_2_) and antisense d(G_2_C_4_) repeats. These RNA transcripts, which can also adopt non-canonical structures, accumulate as toxic aggregates in the nucleus and can sequester RNA-binding proteins (1,17–20). Additionally, repeat-containing RNAs can act as a substrate for repeat-associated (RAN) translation, which produces toxic dipeptide-repeat proteins (DPR) (19,21–23). Recent studies also suggest that non-canonical structures in the DNA repeat-region promote formation of double strand breaks, which coupled with impaired DNA repair pathways lead to apoptosis of neuronal cells (24–26). Different mechanisms, most likely, conspire in advancing neurodegeneration caused by the *C9orf72* repeat expansion.

G-quadruplexes are four-stranded DNA and RNA structures, which consist of stacked layers of G-quartets. Each G-quartet is formed by four coplanar guanine residues held together by Hoogsteen-type hydrogen bonds and stabilized by a central cation. Topologies of the G-quadruplexes can be broadly classified according to orientation of the four strands as antiparallel, parallel, or hybrid (3 + 1). Accompanied by different molecularity, the number of G-quartets, groove dimensions, and types of loops, G-quadruplexes can achieve tremendous structural diversity. Several different G-quadruplex species often coexist in solution which represents a great challenge for structural studies. Incorporation of a C2′-fluorinated guanosine analogue, which preferentially adopts an *anti* conformation, can drive the folding of a G-rich oligonucleotide towards a particular topology (27,28). Similarly, incorporation of 8-bromo-2′-deoxyoguanosine (8Br-dG) which adopts a *syn* glycosidic conformation has been exploited to stabilize a single G-quadruplex species in NMR structural studies (29–31).

Due to potentially high relevance of non-canonical structures adopted by d(G_4_C_2_) repeats in the mechanism triggering devastating neurodegenerative disorders, we explored structural characteristics of oligonucleotides with four d(G_4_C_2_) repeats. Four repeats were chosen as the shortest DNA sequence that can adopt a unimolecular G-quadruplex structure. Earlier studies have shown that a four d(G_4_C_2_) repeat DNA oligonucleotide forms a mixture of mostly antiparallel G-quadruplexes in the presence of K^+^ ions (7,8). We examined by ^1^H NMR and CD spectroscopy different DNA oligonucleotides, which contained four d(G_4_C_2_) repeats and were extended or modified at the 5′- and/or 3′-ends (32). It turned out that wt22, d[(G_4_C_2_)_3_G_4_], showed the most favorable ^1^H NMR spectrum and was chosen for further study. Preliminary analysis of the 2D NMR and CD spectra showed that two distinct antiparallel G-quadruplexes were formed at higher population in addition to (several) other G-quadruplexes with lower population (32,33). However, coexistence of several species made structural analysis of wt22 difficult. Single substitutions of dG with its analogue 8Br-dG can lead to stabilization of anticipated structure and were introduced at several positions in wt22. Oligonucleotide sl21, with dG to 8Br-dG substitution at position 21, was chosen for further scrutiny based on the favorable ^1^H NMR and CD spectral characteristics and spectral similarity to wt22 (32). Oligonucleotide sl21 forms two distinct antiparallel G-quadruplexes which coexist in solution (33). The G-quadruplex species named NAN which is favored thermodynamically is a very stable and compact structure with four G-quartets and three lateral loops. One of the cytosine residues in each lateral C–C loop is stacked over the nearby G-quartet (30). Herein, we used solution NMR to determine the high-resolution structure of the second G-quadruplex species adopted by sl21, which corresponds to one of the major G-quadruplex structures adopted by its natural analogue wt22. This G-quadruplex species named AQU is favored kinetically and exhibits high thermal and long-term stability. Interestingly, it is stabilized under slightly acidic conditions. High-resolution structure of the G-quadruplex determined in this work contributes to our understanding of the structural diversity of G-quadruplexes and shows that pH can fine-tune G-quadruplex stability by affecting interactions between loop residues. Insights into the structural characteristics of d(G_4_C_2_) repeats uncover structural details which tune polymorphic behavior of G-rich repeats in general, and might facilitate the design of small molecules that can be used to modulate aberrant transcription of *C9orf72* gene that has been linked to ALS and FTD in particular.

## MATERIALS AND METHODS

### Sample preparation

Oligonucleotides were synthesized on a K&A Laborgeraete GbR DNA/RNA Synthesizer H-8 using standard phosphoramidite chemistry in DMT-on mode, purified by reverse-phase HPLC, and desalted on a Sephadex G25 column as previously described (33). Details on folding of the samples are given in the Supplementary Data. Concentration of samples was determined by UV absorption at 260 nm using Spectrophotometer Varian CARY-100 BIO UV–VIS. Extinction coefficients of the oligonucleotides with standard dG and dC residues were determined by the nearest neighbor method. Extinction coefficients of the oligonucleotides with non-standard bases were determined by the base composition method using values of 11.3 and 5.7 l mol^−1^ cm^−1^ for 8Br-dG and 5Me-dC, respectively.

### NMR experiments and restraints

NMR experiments were performed on the 600 and 800 MHz Agilent-Varian NMR spectrometers equipped with triple resonance HCN cold probe or OneNMR probe (for variable temperature experiments). 1D ^1^H NMR spectra were acquired with the spectral width of 12 kHz (on 600 MHz) or 14 kHz (on 800 MHz), 128 scans and 1.5 s recycle delay. NOESY spectra were acquired with the spectral width of 12 kHz (on 600 MHz) or 14 kHz (on 800 MHz) in both dimensions, 2048 complex points in *t*_2_, recycle delay of 1.5 s, 320 *t*_1_ increments and 40 scans per *t*_1_ increment. The spectra were apodized with a cosine squared function and zero filled twice before the Fourier transform. Double-pulsed field gradient spin echo (DPFGSE) pulse sequence was used to suppress the water signal. The 2D spectra were acquired at 5, 25, 35 and 45°C with samples in 90% H_2_O, 10% ^2^H_2_O and 100% ^2^H_2_O. The standard homonuclear 2D NMR experiments recorded at 25°C in 100% ^2^H_2_O including 2D DQF-COSY, TOCSY (20, 40, 60 and 80 ms mixing time) and NOESY (80, 150 and 250 ms mixing time), were used to assign the non-exchangeable protons. Exchangeable proton resonances were assigned using 2D NOESY (60, 100, 150, 250, 300, 400 ms mixing time) recorded in 90% H_2_O, 10% ^2^H_2_O at 25°C. The details on NMR restraints used for structure calculation, assignment of cytosine residues and additional comments on the assignment of sugar puckering are given in the Supplementary Data. NMR spectra were processed and analyzed using VNMRJ (Agilent-Varian) and Sparky (UCSF) software.

### Structure calculations

Structure calculations were performed with the AMBER 14 software (34) using the parmbsc0 force-field (35) with parmχOL4 (36) and parmϵ/ζOL1 (37) modifications. Calculations were started from an initial extended structure of oligonucleotide d[(G_4_C_2_)_3_GG^Br^GG], created with LEAP module of the AMBER 14 program. Hydrogen bonds, planarity, chirality and chi torsion angle restraints were included over three steps to generate a topology consistent with the NMR data. In the next step, all NOE and torsion angle restraints were included to generate a total of 100 structures calculated in 130 ps of NMR restrained simulated annealing (SA) simulations (details on SA can be found in the Supplementary Data). Twenty structures with the lowest energy were chosen and energy minimized. The final ten structures were chosen based on the lowest violations of NMR restraints from a family of twenty lowest-energy structures. Figures were visualized and prepared with UCSF Chimera software (38).

## RESULTS

### Structural polymorphism of d[(G_4_C_2_)_3_G_4_] is reduced by combining the appropriate folding conditions and restraining the glycosidic conformation of a single guanine residue

**Figure 1.**
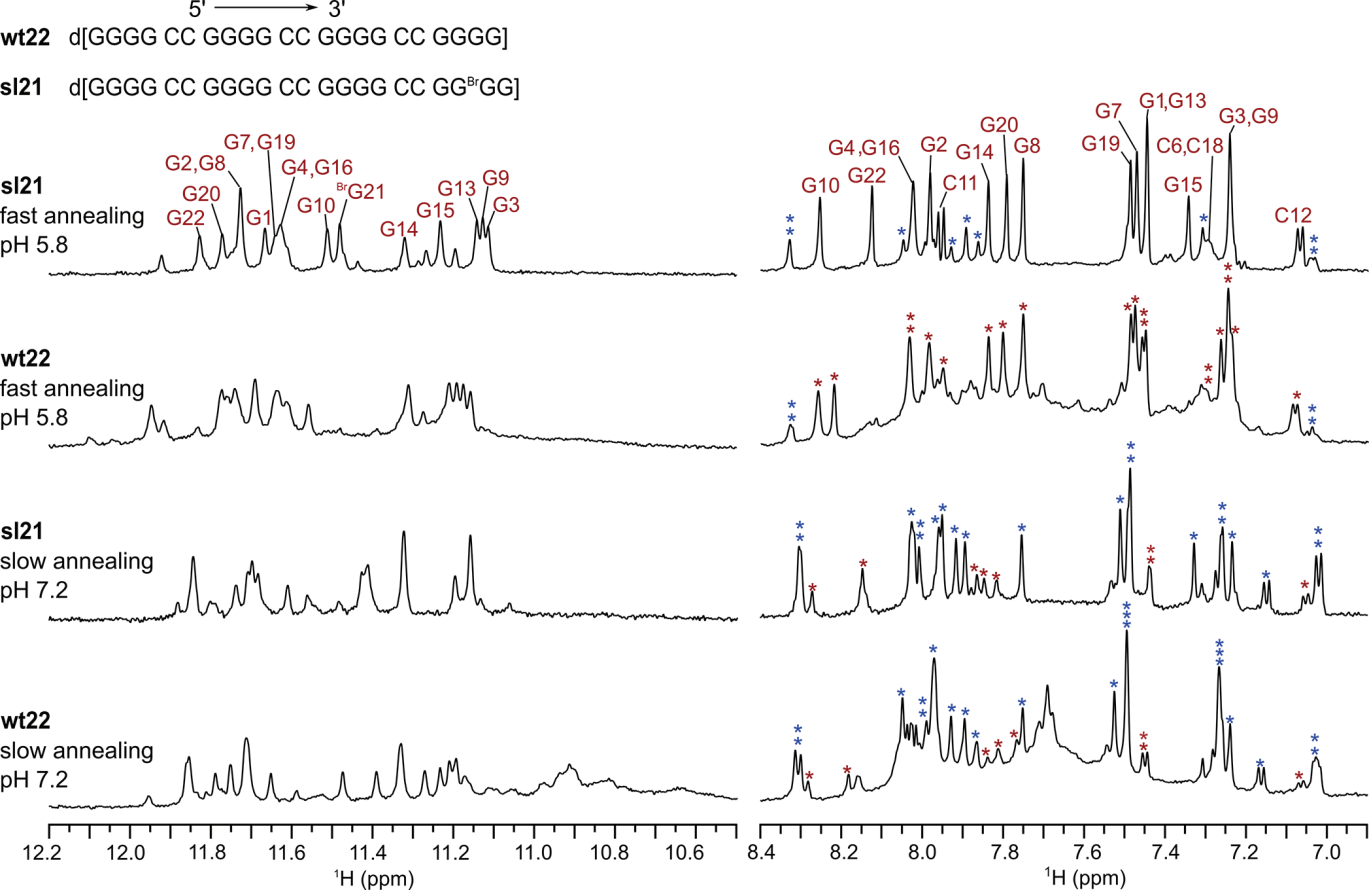
Sequence and the ^1^H NMR spectra of wt22 and sl21 in the presence of K^+^ ions. Assignment of imino and aromatic resonances of sl21 corresponding to AQU is shown above the corresponding signals. Signals in the aromatic region marked with red and blue stars indicate resolved signals corresponding to AQU and NAN, respectively. Oligonucleotide name and folding conditions (fast annealing in the presence of 30 mM KCl at pH 5.8 or slow annealing in the presence of 120 mM KCl at pH 7.2) are indicated next to individual spectrum. Spectra were recorded at 600 MHz, 25°C in 10% ^2^H_2_O, 90% H_2_O, 30 or 120 mM KCl, pH 5.8 or 7.2 (20 mM K-phosphate buffer) and oligonucleotide concentrations between 0.05 and 0.15 mM.

A truncated four d(G_4_C_2_)-repeat oligonucleotide named wt22, d[(G_4_C_2_)_3_G_4_], forms multiple structures in the presence of K^+^ ions. Our preliminary NMR study showed that two G-quadruplexes are formed at higher population in addition to (several) G-quadruplex structures with lower population (32,33). Substitution of dG with 8-bromo-2′-deoxyguanosine (8Br-dG) at position 21 decreases formation of low-populated structures, which results in considerably improved NMR spectral characteristics of oligonucleotide d[(G_4_C_2_)_3_GG^Br^GG] named sl21, compared to its natural counterpart wt22 (Figure 1). Two sets of sharp signals in the ^1^H NMR spectrum of sl21 indicate coexistence of two G-quadruplex structures. Importantly, the signals in the ^1^H NMR spectrum of sl21 exhibit similar intensities and chemical shifts as the major sharp signals of wt22. This indicates that the two structures stabilized by introduction of 8Br-dG are highly-populated in wt22 (Figure 1). Interestingly, the relative populations at which the G-quadruplex structures are formed by sl21 (and wt22) depend on two folding conditions: solution pH and the rate of cooling in the presence of K^+^ ions after thermal denaturation by heating to 90°C (Figure 1). When sl21 is cooled slowly (in 16 h) to 25°C at pH 7.2, a G-quadruplex structure denoted NAN (for neutral and annealing) is favored with a population of around 70%. Its high-resolution structure was reported by us earlier (30). In comparison, when sl21 is cooled quickly (in minutes) to 0°C at pH 5.8, a distinct G-quadruplex structure denoted AQU (for acidic and quenching) is favored with a population of ∼80%, while NAN is present as a minor species (20%). Noteworthy, concentration of KCl does not influence the relative ratio between the populations of AQU and NAN. However, when sl21 is folded with fast annealing at pH 5.8, the intensity of broad and poorly defined signals is increased at 120 mM compared to 30 mM KCl. This suggests that the population of additional unidentified structures or possibly aggregated forms is increased at higher KCl concentration. In comparison, the ^1^H NMR spectra of sl21 folded with slow annealing at pH 7.2 in the presence of 120 or 30 mM KCl are very similar (Supplementary Figure S1). No changes in the ^1^H NMR spectra of sl21 folded into mostly NAN or mostly AQU are observed even after several months of storage at 25°C, indicating prolonged stability of both structures (Supplementary Figure S3 in (33)). Notably, the identical pattern of cross-peaks in NOESY spectra (Supplementary Figure S2; see Supplementary Figure S2 in ref. (33) for comparison of additional regions of NOESY spectra) suggests that the structure of AQU adopted by sl21 and wt22 is expected to be very similar. Insights based on unequivocal spectral assignment of H1 and H8 protons of sl21 and CD spectrum which displayed a minimum at 260 nm and maxima at 245 and 295 nm, indicated that AQU is an antiparallel G-quadruplex with four G-quartets (33).

### Assignment of NMR spectra of AQU uncovers dynamic equilibrium of C–C loops

Fast annealing of aqueous solution of sl21 in the presence of 30 mM KCl and pH 5.8 results in formation of a G-quadruplex structure named AQU with a population of 80%, which has enabled determination of its high-resolution structure. Assignment of ^1^H resonances of sl21 corresponding to AQU (Supplementary Table S1) was achieved using standard 1D and 2D NMR experiments including NOESY (τ_m_ from 60 to 400 ms), TOCSY, DQF-COSY and ^13^C-HSQC. Guanine imino (H1) and aromatic (H8) resonances were unambiguously assigned by ^15^N- and ^13^C-edited HSQC spectra acquired on partially (8%) residue-specifically ^15^N- and ^13^C-labeled sl21 (33). Strong intraresidual H1′–H8 cross-peaks in NOESY spectrum (τ_m_ = 80 ms) indicate that residues G1, G3, G7, G9, G13, G15 and G19, together with ^Br^G21, adopt predominately *syn* conformations around the glycosidic bonds (Supplementary Figure S3), while the rest of the guanine residues adopt *anti* conformation. Sequential connectivities were followed easily in the H6/H8-H2′/H2″ region of NOESY (τ_m_ = 300 ms) spectrum and displayed expected interruptions in H6/H8(*n*+1)-H2′/2″(*n*) connectivities at the 5′ *anti*-3′ *syn* steps (Supplementary Figure S4). Sequential connectivities could also be traced in the H6/H8-H3′ region, whereas analysis of the H6/H8-H1′ region of NOESY spectra was complicated due to the substantial signal overlap (Supplementary Figures S5 and S6, additional comments on the assignment can be found in the Supplementary Data). Perusal of DQF-COSY and TOCSY spectra demonstrated that all the guanine residues and C12 exhibit predominately S-type sugar conformation, while C11 displays a preference for N-type sugar puckering. The conformation of sugar moieties of C5, C6, C17 and C18 could not be determined due to signal broadening (*vide infra*). Assignments of H6 resonances of C11 and C12 were obtained through sequential walk in NOESY spectra and were corroborated by the substitution of dC with 5-methyl-2′-deoxycytidine (5Me-dC). Interestingly, only three strong H5-H6 cross-peaks were observed in the NOESY and TOCSY spectra of sl21, although there are six cytosine residues in the sequence (Supplementary Figure S3). Two out of the three resolved H5–H6 cross-peaks correspond to C11 and C12. Assignment of the third H5-H6 cross-peak was complicated due to signal broadening and spectral overlap. Assignment was achieved with the help of oligonucleotides, in which cytosine residues at positions 5, 6, 17 and 18 were substituted one-by-one or in pairs with 5Me-dC (Supplementary Table S2). Comparison of NOESY spectra of 5Me-dC substituted oligonucleotides showed that an apparently single H5–H6 cross-peak corresponds to C6 and C18 (Supplementary Figure S7). The H5–H6 cross-peaks corresponding to C5 and C17 are broadened to the baseline in NOESY and TOCSY spectra (Supplementary Figure S3). Residues C5 and C17 as well as residues C6 and C18 exhibit isochronous proton chemical shifts and identical cross-peak patterns in 2D spectra of sl21. This points to a C2-symmetry of C5–C6 and C17–C18 loops, which was corroborated by comparison of NOESY spectra of sl21 analogues with dC to 5Me-dC substitutions (Supplementary Figures S8 and S9). Many cross-peaks involving protons of C5, C6, C17 and C18 were broadened in the NOESY spectra recorded at 25°C, which is consistent with the conformational motions that are intermediate on the ^1^H NMR chemical shift time-scale i.e. in the micro-to-millisecond range. These motions reach a fast exchange regime at 45°C, leading to sharpening of cross-peaks (Supplementary Figure S10). A slow exchange regime that would exhibit distinct signals for the individual slowly exchanging conformations was not reached even at −10°C.

**Figure 2.**
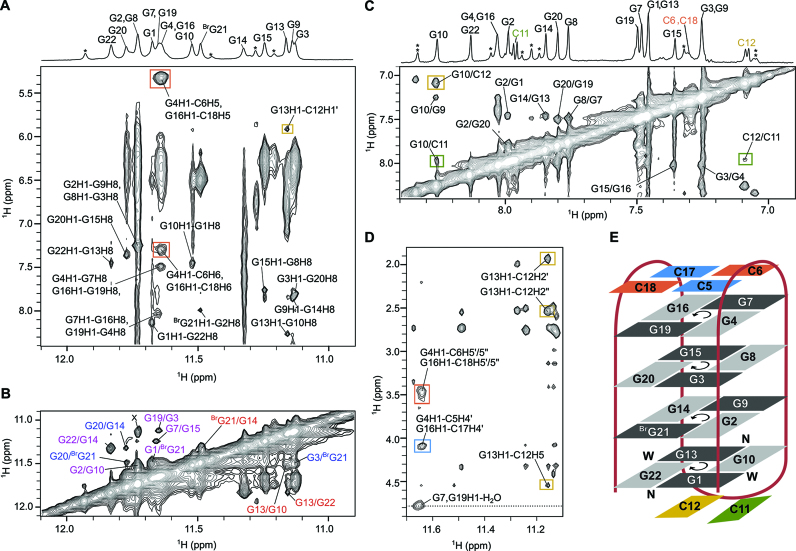
NOESY spectra of sl21 and the topology of AQU. (**A**) The imino-aromatic region of NOESY (τ_m_ = 300 ms) spectrum showing typical H1–H8 correlations between residues in G-quartets. (**B**) The imino-imino region of NOESY (τ_m_ = 300 ms) spectrum. The cross-peaks between imino protons of residues in adjacent G-quartets are shown in magenta (between inner and outer G-quartet) and blue (between two inner G-quartets). Intraquartet cross-peaks are shown in red. Cross-peak marked with an X could be interpreted as G2/G3, G2/G9, G8/G9 and/or G8/G3. (**C**) The aromatic-aromatic region of NOESY (τ_m_ = 400 ms) spectrum. (**D**) Part of the imino-sugar region of NOESY (τ_m_ = 400 ms) spectrum. 1D ^1^H NMR spectrum with assignments of imino and aromatic protons of AQU adopted by sl21 are shown above the 2D plots. Signals corresponding to NAN, which is present as a minor species, are indicated by stars. In all panels, NOE cross-peaks involving cytosine residues are marked with rectangles in blue (C5 and C17), orange (C6 and C18), green (C11) and yellow (C12). (**E**) The topology of AQU. Anticlockwise and clockwise directionalities of hydrogen bonds within G-quartets are marked. Guanine residues in *syn* and *anti* glycosidic conformations are depicted as dark and light gray rectangles, respectively. Letters W and N denote wide and narrow grooves, respectively. NOESY spectra were recorded at 600 MHz, 25°C in 10% ^2^H_2_O, 90% H_2_O, 30 mM KCl, pH 5.8 (20 mM K-phosphate buffer) and oligonucleotide concentration of 1.0 mM.

Cross-peaks between H1 and H8 of adjacent residues in G-quartets in NOESY (τ_m_ = 300 ms) spectrum (Figure 2A) revealed that AQU is composed of the following four G-quartets: G1:G22:G13:G10, G2:G9:G14:^Br^G21, G3:G20:G15:G8 and G4:G7:G16:G19, which display alternating clockwise–anticlockwise–clockwise–anticlockwise directionality of hydrogen bonds. Relative position of the G-quartets in the structure was determined through examination of inter-quartet H1–H1 cross-peaks (Figure 2B). Moreover, H8–H8 NOE cross-peaks suggest efficient stacking between the sequential guanine residues of the inner and outer G-quartets (Figure 2C). In addition to the expected sequential NOEs, several unusual NOE contacts were observed between the cytosine residues in the loops and the guanine residues in the nearby G-quartets (Figure 2 and Supplementary Figure S11). In the C11–C12 loop, C12 sugar and aromatic H5 protons exhibit NOE contacts with H1 of G13. In addition, large number of NOE contacts are visible between C12 and G10, whereas there are only two NOE contacts between C11 and G10. Together, these NOE contacts place C12, rather than C11, over the G1:G22:G13:G10 quartet where it could get involved in stacking interactions. In the lateral loop comprised of C5 and C6, H5 and H6 of C6 display NOE contacts with H1 of G4. Analogous NOE contacts are observed between C18 and G16, suggesting that both C18 and C6 are stacked on the G4:G7:G16:G19 quartet. Broadening of the signals corresponding to aromatic protons of C5 and C17 correlates with lack of (expected) long-range NOE contacts. However, we observed many sequential NOE cross-peaks involving the sugar protons of C5 and C17, which suggests stacking between sequential residues at the G4–C5 and G16–C17 steps. In summary, our NMR data show that AQU is an antiparallel G-quadruplex consisting of four G-quartets, where every strand is antiparallel with respect to the adjacent strands and exhibits an alternating *syn–anti* progression of glycosidic conformation of the guanine residues. The antiparallel strands in AQU form alternating narrow and wide grooves. Two of the lateral loops, C5–C6 and C17–C18, span wide grooves, whereas the C11–C12 loop spans a narrow groove (Figure 2E).

### Antiparallel G-quadruplex with four G-quartets and well-defined lateral loops is stabilized by stacking of C•C base pairs

**Figure 3.**
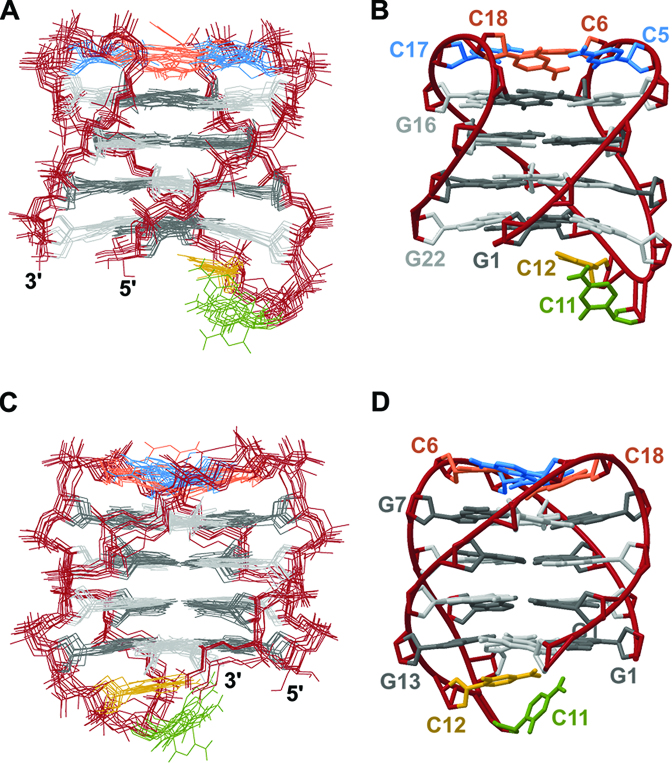
Solution structure of AQU adopted by sl21. View into the narrow (**A**) and wide (**C**) grooves of ten superimposed structures with the lowest energy and restraint violations. View into the narrow (**B**) and wide (**D**) grooves of a ribbon presentation of the representative structure. For clarity, cytosine residues are depicted in blue (C5 and C17), orange (C6 and C18), green (C11) and yellow (C12). Guanine residues in *syn* and *anti* glycosidic conformation are shown in dark and light gray color, respectively. Backbone atoms (A) and ribbon (B) are shown in dark red.

Solution structure of AQU adopted by sl21 was calculated using 310 NOE-derived distance restraints, together with 36 torsion angle restraints, 32 hydrogen bond restraints and 48 planarity restraints (Table 1). Planarity restraints were omitted in the last 30 ps of simulated annealing and during energy minimization. Ten structures that display the lowest energies and the smallest violations of experimental restraints were chosen from a set of 100 calculated structures (Figure 3). The structure of AQU is well-defined with the overall pairwise heavy atom RMSD of 1.0 Å (Table 1). Pseudo-C2 axis of symmetry which runs through the central cavity of the G-quartet core is broken down by the C11–C12 loop and by the presence of 8Br-dG residue at position 21.

**Table 1.** Structural statistics for AQU adopted by sl21

**NMR restraints**	
NOE-derived distance restraints	
Total	310
Intra-residue	197
Inter-residue	113
Sequential	93
Long-range	20
Torsion angle restraints	
Chi (χ)	18
Sugar puckering	18
Hydrogen bond restraints	32
G-quartet planarity restraints*	48
**Structure statistics**	
Violations	
Mean NOE restraint violation (Å)	0.10 ± 0.03
Max. NOE restraint violation (Å)	0.18
Mean torsion angle restraint violation (°)	3.32 ± 0.94
Max. torsion angle restraint violation (°)	6.20
Deviations from idealized geometry	
Bonds (Å)	0.012 ± 0.000
Angles (°)	2.45 ± 0.04
**Pairwise heavy atom RMSD (Å)**	
Overall	1.00 ± 0.25
G-quartets	0.87 ± 0.25
G-quartets and C11–C12	0.93 ± 0.25
G-quartets with C5–C6 and C17–C18	0.94 ± 0.26

*Planarity restraints were omitted in the last 30 ps of SA and during energy minimization.

**Figure 4.**
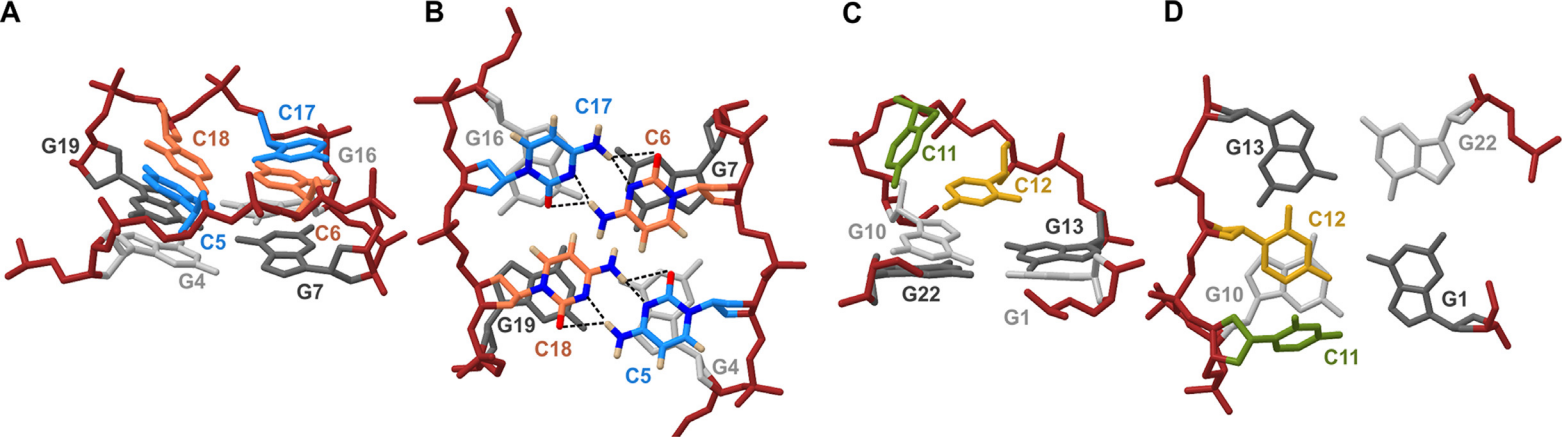
Structure of the lateral loops in AQU. (**A**) Side view and (**B**) bird's eye view of the C5–C6 and C17–C18 loops stacked on the G4:G7:G16:G19 quartet. Possible hydrogen bonds within the C5•C18 and C6•C17 base pairs are depicted by the dashed lines. Oxygen, nitrogen, and hydrogen atoms of cytosine residues in panel B are shown in red, blue and beige, respectively. (**C**) Side view and (**D**) bird's eye view of the C11–C12 loop above the G1:G22:G13:G10 quartet. For clarity, cytosine residues are depicted in blue (C5 and C17), orange (C6 and C18), green (C11) and yellow (C12). Guanine residues in *syn* and *anti* glycosidic conformation are shown in dark and light gray color, respectively. Backbone atoms are shown in dark red.

In the lateral loop comprised of C5 and C6, two cytosine residues are nearly coplanar and stacked on the residues G4 and G7 of the nearby G4:G7:G16:G19 quartet, respectively (Figure 4A). On the opposite side of G4:G7:G16:G19 quartet, lateral loop comprised of C17 and C18 adopts a very similar structure, with residues C17 and C18 stacked on G16 and G19, respectively. The cytosine residues stacked on the G4:G7:G16:G19 quartet are oriented in way which suggests that they form C5•C18 and C6•C17 base pairs (Figure 4B). In the C•C base pairs, distances between the amino H4 of one residue and N3 as well as O2 of another residue are short and consistent with the formation of hydrogen bonds (around 2 and 2.6 Å, respectively). This observation suggests that the C•C base pairs are likely to be involved in an equilibrium between symmetric N3-amino and symmetric carbonyl-amino geometry. Although isochronous chemical shifts and identical NOE patterns indicate that the C5–C6 and C17–C18 loops are highly symmetric, relative positions of the C5•C18 and C6•C17 base pairs with respect to the nearby G4:G7:G16:G19 quartet are not identical (Figure 4A and B). Five out of ten structures in the structural ensemble display C6•C17 base pair closer to the G4:G7:G16:G19 quartet, while four structures show C5•C18 base pair closer to the G-quartet (Supplementary Figure S12). In one out of ten structures, C5 and C18 form a base pair, while C6 is placed above the plane formed by C5, C17 and C18 (Supplementary Figure S12). Four hydrogen-bonded amino (H4) protons are present in C5•C18 and C6•C17 base pairs, however we did not observe the corresponding signals in the ^1^H NMR spectra. This is most likely due to intermediate-exchange regime between the different hydrogen-bonding patterns within the C5•C18 and C6•C17 base pairs and different relative position of the base pairs with respect to the nearby G-quartet, which were established through structure calculations. In the lateral loop comprised of C11 and C12, base moiety of C12 is parallel to the G1:G22:G13:G10 quartet and lies above G10 and G13 (Figure 4C). Shielding due to ring current effects is in agreement with the observed upfield chemical shift of C12H5 (Supplementary Table S1, δ 4.51 ppm at 25°C). Pyrimidine moiety of C11 is tilted with respect to the G1:G22:G13:G10 quartet at approximately 45° and is placed above C12 with its aromatic protons turned away from the G-quartet (Figure 4D).

**Figure 5.**
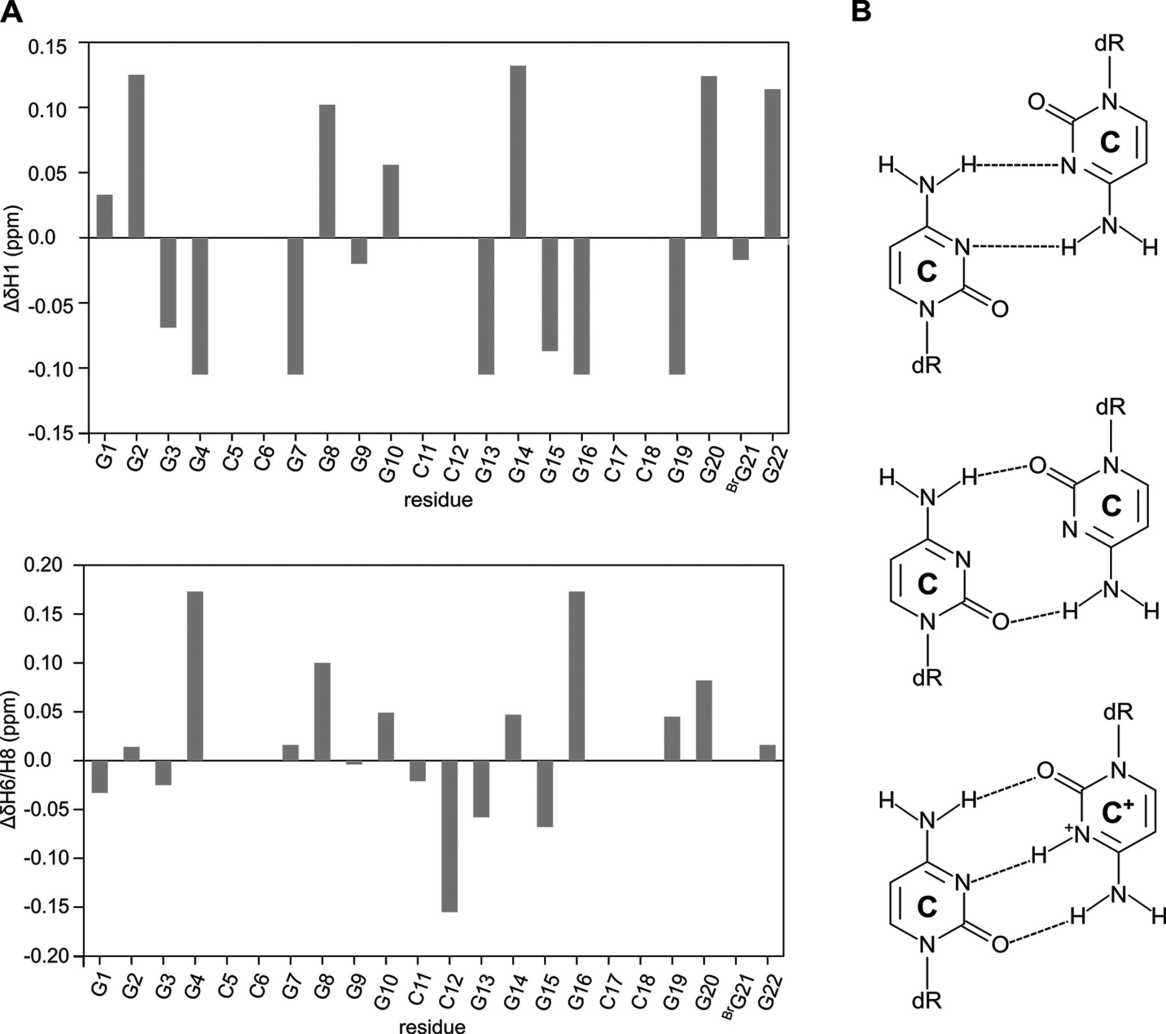
Chemical shift (de)shielding of individual residues of AQU adopted by sl21 at alkaline relative to acidic pH conditions. (**A**) Differences in the ^1^H NMR chemical shifts of H1 and H6/H8 signals at pH 9.4 and 4.0 (Δδ = δ9.4-δ4.0) at 25°C. Data for aromatic protons of residues C5, C6, C17 and C18 are missing because the corresponding signals were not resolved in the ^1^H NMR spectra. (**B**) Schematic representation of C•C base pairs in N3-amino geometry (top), carbonyl-amino geometry (middle), and C^+^•C base pair (bottom).

In addition to being favored kinetically, AQU is preferentially formed under acidic conditions. In our earlier study (33) we showed that the population of AQU formed by sl21 increases from 30% at pH 7.2 to 50% at pH 5.8, when sl21 is folded with slow annealing. When folded with fast annealing, population of AQU increases from 67% at pH 7.2 to 80% at pH 5.8. Importantly, relative populations of AQU (80%) and NAN (20%) are not changed when pH is varied between 4.0 and 9.4 after the steady state has been established. However, variation of pH in the above range causes broadening and chemical shift differences in some of the H1 and H8 signals corresponding to AQU (Figure 5A and Supplementary Figure S13). Signals which correspond to the overlapped H8 of G4 and G16 are shifted by more than Δδ 0.15 ppm downfield upon change of pH from 4.0 to 9.4, which suggests that the cytosine residues involved in C5•C18 and C6•C17 base pairs (stacked on the G4:G7:G16:G19 quartet) undergo protonation in the acidic solution conditions. Notably, protonation of cytosine residues can be expected considering that the p*K*_a_ of N3 in free cytosine is ∼4.2 (39) and shifts towards higher values when protonation is coupled to the formation of C^+^•C base pair (40). The overlapped signal corresponding to the H1 of residues G4, G7, G16 and G19 is broadened at pH 6.0 and disappears in the spectra recorded between pH 6.4 and 8.3. The signal reappears at 9.4, shifted by Δδ 0.1 ppm upfield compared its position in the spectrum recorded at pH 4.0. Spectral broadening between pH 6.4 and 8.3 is observed also for H8 signals of G4 and G16 and to a lesser extent for H8 signals of G3, G7, G8, G15 and G19 and the H1 of G3 and G15 (Supplementary Figure S13). These data suggest that the C5•C18 or C6•C17 base pairs are involved in the dynamic equilibrium between protonated C^+^•C and unprotonated C•C state. This equilibrium is intermediate on the ^1^H NMR chemical shift time-scale and might be coupled to an increased dynamic of the nearby G-quartets. Signals sharpen in the ^1^H NMR spectrum recorded at pH 9.4, suggesting that equilibrium is shifted towards unprotonated C•C state under the alkaline solution conditions. Notably, C^+^•C base pairs are stabilized with additional hydrogen bonds compared to unprotonated C•C base pairs. Two additional hydrogen bonds and possibly more favorable stacking interaction upon protonation of C5•C18 and C6•C17 base pairs provide structural rationalization for increased population of AQU at slightly acidic conditions as compared to the neutral solution conditions. The signal of C12H6 is shifted upfield by Δδ 0.15 ppm upon pH increase from 4.0 to 6.0, but displays almost no change upon increase of pH from 6.0 and 9.4. In comparison, C11H6 signal changes by less than Δδ 0.04 ppm between pH 4.0 and 9.4. The chemical shift changes indicate that only residue C12 within the C11-C12 loop is significantly protonated at pH value of 4.0.

### Insights into (non)interconversion between the two G-quadruplex structures adopted by sl21

**Figure 6.**
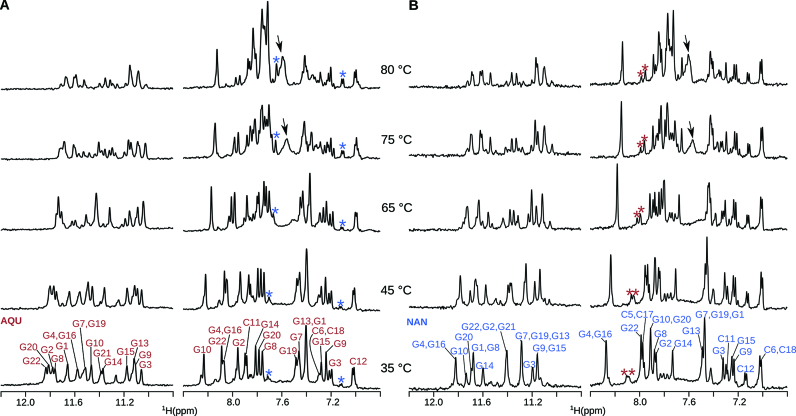
Imino and aromatic regions of the variable temperature ^1^H NMR spectra. (**A**) Variable temperature ^1^H NMR spectra of sl21 (80% AQU and 20% NAN) with the assignment of AQU shown at 35°C. (**B**) Variable temperature ^1^H NMR spectra of sl21 (30% AQU and 70% NAN) with the assignment of NAN shown at 35°C. Spectra were recorded at 600 MHz in 10% ^2^H_2_O, 90% H_2_O, 10 mM KCl, pH 7.2 (20 mM K-phosphate buffer) and oligonucleotide concentration of 0.1 mM. Stars indicate resolved signals of minor species AQU (red) and NAN (blue). Arrow indicates signals of unfolded sl21.

Variable temperature ^1^H NMR experiments were used to gain insight into partial local melting and slow equilibrium of the two G-quadruplex structures formed by sl21, AQU and NAN. Variable temperature ^1^H NMR experiments performed on sl21, folded into 80% AQU and 20% NAN, in the presence of 30 mM KCl at pH 5.8 showed that sl21 does not unfold up to 80°C (Supplementary Figure S14). In order to facilitate G-quadruplex unfolding at lower temperature, the variable temperature experiment was repeated in the presence of lower, 10 mM concentration of K^+^ ions (Figure 6). In addition, experiment was performed separately on two different samples of sl21, which differed in the ratio between the starting populations of AQU and NAN. The first sample of sl21 was folded with fast annealing at pH 5.8 and contained 80% AQU and 20% NAN (Figure 6A). The signals in the ^1^H NMR spectra corresponding to the imino and aromatic protons of AQU display minimal changes as the temperature is increased from 35 up to 65°C (Figure 6A). When the temperature is increased to 75°C, we can observe new signals only in the aromatic region (indicated by an arrow in Figure 6A). These signals are reminiscent of the aromatic signals corresponding to unfolded sl21 (Supplementary Figure S15). This data might indicate that AQU is starting to unfold at 75°C. In addition, the relative population of NAN with respect to AQU is slightly increased at 75°C compared to 35°C. At 80°C, most of sl21 remains folded as evidenced by the presence of G-quadruplex imino and aromatic signals in the spectrum. However, NAN becomes the predominant species at 80°C with relative populations of AQU and NAN at around 30% and 70%, respectively. The second sample of sl21 was folded with slow annealing at pH 7.2 and initially (at 35°C) contained 70% NAN and 30% AQU (Figure 6B). Spectra recorded between 35 and 80°C show no indication of change in the relative populations of NAN with respect to AQU (Figure 6B). Our variable temperature NMR data suggest that AQU and NAN are two folded states of sl21 separated by a high-energy barrier that prevents their inter-conversion. Consequently, AQU and NAN inter-convert only through unfolded state. When the temperature is increased starting from non-equilibrium populations (80% AQU and 20% NAN), the population of the more stable structure (NAN) increases as the less stable (AQU) structure melts. However, both G-quadruplex structures have very high thermal stability; AQU unfolds between 75 and 80°C, whereas NAN does not unfold even at 80°C.

## DISCUSSION

Exploration of the structural characteristics of d(G_4_C_2_) repeat has its merits due to potential roles of the non-canonical structures formed within the expanded d(G_4_C_2_)•(G_2_C_4_) repeats in the disease mechanism of ALS and FTD. First, formation of G-quadruplexes has been proposed as a cause of genetic instability, which is the most common genetic cause of ALS and FTD. Second, G-quadruplexes (and R-loops) which form in the non-coding region of *C9orf72* lead to aberrant gene transcription with a myriad of downstream pathological consequences resulting in the development of ALS and FTD. Here, we present a high-resolution structure of one of the major G-quadruplex structures adopted by the oligonucleotide d[(G_4_C_2_)_3_G_4_], which represents the shortest model of the d(G_4_C_2_) repeats that can form a unimolecular G-quadruplex. Oligonucleotide d[(G_4_C_2_)_3_G_4_] folds into multiple G-quadruplex structures in the presence of K^+^ ions. Two distinct antiparallel G-quadruplexes together account for ∼80% of the total G-quadruplexes formed by the wild type oligonucleotide, while the remaining 20% consists of unidentified or, possibly, aggregated forms. Introduction of 8Br-dG residue at position 21 in oligonucleotide sl21, d[(G_4_C_2_)_3_GG^Br^GG], enabled us to determine the 3D structures of the two major G-quadruplexes. Relative populations at which the two G-quadruplex species are formed by sl21 depend on pH during folding and a rate at which the solution is cooled after heating to 90°C. When sl21 is cooled slowly at pH 7.2, G-quadruplex named NAN (for neutral and annealing) forms at ∼70%, while a distinct G-quadruplex denoted AQU forms at 30%. Ratio of AQU and NAN populations is reversed if solution of sl21 is cooled quickly at pH 7.2, resulting in formation of AQU with population of 67% and NAN at 33%. Further increase in the population of AQU with respect to NAN is achieved when solution of sl21 is cooled quickly under slightly acidic solution conditions (pH 5.8). In this case, AQU (for acidic and quenching) is favored at around 80%, while NAN is present as a minor species at 20%. Remarkably, once stationary state is reached, the ratio between the populations of AQU (80%) and NAN (20%) does not change even under alkaline solution conditions or after months of storage at room temperature. In fact, the two G-quadruplexes inter-convert through unfolded state only. Both structures exhibit high thermal stability, which suggests that when AQU and NAN form *in vivo*, specialized helicases (41) would be needed to unwind them.

**Figure 7.**
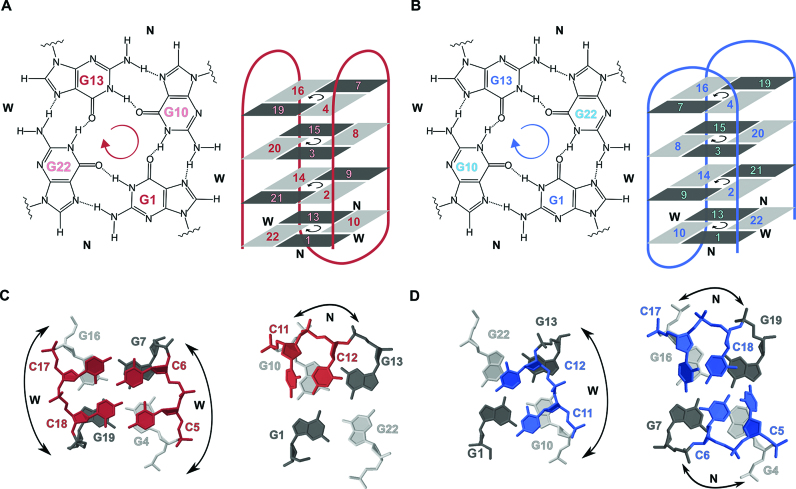
Comparison of the structural features of AQU (red) and NAN (blue). (**A**) G1:G22:G13:G10 quartet and topology of AQU. (**B**) G1:G10:G13:G22 quartet and topology of NAN. Structure of the lateral loops in AQU (**C**) and NAN (**D**). In panels A and B, anticlockwise and clockwise directionalities of hydrogen bonds within the G-quartets are marked. Guanine residues in *syn* and *anti* glycosidic conformations are depicted as dark and light gray rectangles, respectively. Letters W and N denote wide and narrow grooves, respectively.

NAN is a compact antiparallel G-quadruplex with four G-quartets and three lateral loops, where one of the cytosine residues in each lateral C–C loop is stacked on the nearby G-quartet (30). The high-resolution structure of AQU determined here uncovers a similar antiparallel type topology with four G-quartets and three lateral loops. When the two G-quadruplexes are placed in a common reference frame proposed by Webba da Silva (42), with orientation of G-rich segments as in Figure 7, the strands and lateral (l) loops in AQU progress anticlockwise (-) and overall topology is (−l,−l,−l). In NAN, the three lateral loops progress in clockwise direction which corresponds to (+l,+l,+l) type topology. Overall, AQU and NAN are similar in terms of the residues which comprise the individual G-quartets, directionality of hydrogen bonds within the G-quartets and *syn* and *anti* conformation of guanine residues along the sequence and in the G-quartets. However, the two structures differ in the donor-acceptor directionalities for each individual hydrogen-bonded pair in the G-quartets. For example, in the G1*(syn)*–G22*(anti)* base pair, the donor of hydrogen bond in AQU is G1, whereas it is G22 in NAN. This reversal affects the width of the grooves defined by a given set of residues, e.g. the G1(*syn)→G22(anti)* is in a narrow groove in AQU, whereas analogous G22*(anti)→*G1(*syn)* pair in NAN is in a wide groove (arrow shows directionality of hydrogen bond). Consequently, the first and the third lateral loops in AQU span wide grooves, whereas the middle loop spans a narrow groove. In comparison, the first and third lateral loops in NAN span narrow grooves and the middle loop spans a wide groove.

Comparison of AQU and NAN reveals that the lateral loops which span grooves of the same type (wide vs. narrow) adopt similar structure (Figure 7C and D). In the lateral C–C loops which span narrow grooves, the second residue is coplanar with a nearby G-quartet. The first residue is placed above the second, and tilted with respect to the G-quartet at an ∼45° angle. In comparison, two cytosine residues are coplanar and stacked over the G-quartet in the lateral loops which span wide grooves. Structural differences between the two types of lateral loops have an important influence on how the loop residues interact when two loops span the edges of the same G-quartet. In NAN, two cytosine residues form a C•C base pair and are stacked over the G-quartet, while the two remaining cytosine residues are turned away from a G-quartet. In AQU, there are two C•C base pairs and four cytosine residues are stacked on the G-quartet. Earlier molecular modeling studies have indicated that short lateral loops (comprised of only two residues) which span wide grooves are under strain and suggested that structures containing two such loops might be distorted and unstable (43). In this regard, AQU represents an unusual example since it is the only G-quadruplex structure where two lateral loops, consisting of only two residues each, span wide grooves. Stabilization due to favorable interactions of loop residues might help to rationalize robustness of the folding topology of AQU. Antiparallel chair-type G-quadruplexes with (–l,–l,–l,) type topology have been observed before (44,45), however AQU is to the best of our knowledge, the first for which a high-resolution structure is available.

AQU is composed of four G-quartets, which are connected by three lateral C–C loops. In the middle C–C loop, the second residue stacks on the G-quartet. On the opposite side of the G-quartet core, two lateral C–C loops span the edges of the same G-quartet. We could expect that the four cytosine residues adopt a C-quartet, a planar arrangement of four cytosine residues connected via cyclic array of hydrogen bonds. However, no C-quartet is present in AQU. Instead, residues of the lateral loops are stacked on the G4:G7:G16:G19 quartet and form two separate, nearly coplanar C5•C18 and C6•C17 base pairs. The C5•C18 and C6•C17 base pairs are dynamic and exchange between symmetric N3-amino geometry, carbonyl-amino geometry and protonated C^+^•C states. In a parallel G-quadruplex adopted by d(TG_3_CG_2_T), which contains repeat sequence from SV40 virus (46), a C-quartet stabilized by hydrogen bonds in amino-O2 geometry is formed between two G-quartets layers. A very similar C-quartet is found in a tetramolecular G-quadruplex adopted by d(TG_2_CG_2_C), which contains two triplet repeats linked to Fragile X syndrome (47). C-quartets exhibit a central cavity with a larger dimension than G-quartets. Under slightly acidic conditions which lead to protonation of cytosine residues, a delocalized positive charge is present in the center of the C-quartet. Under neutral conditions, water molecules which are located in the large central cavity of the C-quartet can bridge interaction between the central divalent cation and cytosine residues, as seen in the crystal structure of a parallel G-quadruplex adopted by d(C_2_A^CNV^KGCGTG_2_) in the presence of Ba^2+^ (48). It should be noted that homopyrimidine quartets (C, T- (49,50)) and U-quartets (51)) have not been observed experimentally in antiparallel G-quadruplexes so far. This may be due to the difference in groove widths between parallel and antiparallel G-quadruplexes. Homopyrimidine quartets which are C4-symmetric might preferentially form in parallel G-quadruplexes where all four grooves are of similar, medium width (P–P distance ∼11 Å). In comparison, antiparallel chair-type G-quadruplexes such as AQU and NAN have two wide (P–P distance ∼15 Å) and two narrow (P–P distance ∼8 Å) grooves. The distance between two pyrimidine residues in a wide groove might be too large to allow them to base pair with each other. Narrow grooves, on the other hand, might be too narrow to allow alignment of two pyrimidine bases in the same plane. Influence of the G-quadruplex groove dimensions on the formation of C-quartets is supported by the molecular dynamics simulations of a G-quadruplex formed by four d(G_4_C_2_) repeats (52). This study shows that three out of four cytosine residues are able to stack on the G-quartet in an antiparallel G-quadruplex (with two wide and two narrow grooves). Structure of AQU reveals how four nearly coplanar cytosine residues can stack with G-quartets within an antiparallel chair-type G-quadruplex fold and form a pseudo C-quartet. Mixed antiparallel G-quadruplexes in which G-quartets alternate and continuously stack with nearly coplanar pairs of C•C base pairs could potentially form in various expansion-prone repetitive sequences, which contain tandem repeats of guanine residues interspersed with cytosines (53–55).

Preference for AQU over NAN under slightly acidic conditions can be rationalized by cytosine protonation which leads to formation of C^+^•C base pairs in AQU, but not in NAN. A more general explanation as to why certain G-quadruplex forming oligonucleotides respond to pH, while others do not, is found by comparing AQU and NAN with two other structures, whose relative populations in solution depend on pH. Truncated human telomeric repeat sequence d[G_3_T_2_AG_3_T_2_AG_3_T_2_AG_2_] folds into two antiparallel basket-type G-quadruplexes with two lateral and a middle diagonal loop, named TD and KDH^+^ (56). The equilibrium is shifted towards KDH^+^ at low pH, driven by a protonation of residue A20 (apparent p*K*_a_ of A20 is 6.5, while p*K*_a_ of free *A* is ∼3.5 (39)). In KDH^+^, residue A20 is in the lateral loop that spans a wide groove and forms a T–A–G base-triad containing four hydrogen-bonds, including a hydrogen bond between protonated A20^+^ and G5. In comparison in TD, A20 is in the lateral loop which spans a narrow groove and forms a less stable A–A–G base-triad with two hydrogen bonds. In both AQU and KDH^+^, the protonated residue is in the lateral loop which spans a wide groove. In this type of lateral loop, two residues can align in the same plane. Such arrangement appears to be favorable for formation of an additional hydrogen bond after protonation. We can anticipate that when a G-quadruplex forming oligonucleotide can adopt two different antiparallel topologies, a topology in which residues such as A or C are in the lateral loop that spans a wide groove would be favored under slightly acidic conditions. This observation can also explain why telomeric-repeat oligonucleotide d[G_3_T_2_AG_3_T_2_AG_3_T_2_AG_3_] converts from hybrid to antiparallel topology upon decrease of pH from 7.0 to 4.5 (57).

Stabilization of AQU in acidic conditions is interesting in view of reports that cellular pH can decrease in neurodegenerative diseases, including ALS (58–60). A decrease in intracellular pH would lead to protonation of C•C base pairs in AQU and stabilize the structure. More persistent G-quadruplex structure would cause increased genetic instability, DNA damage, and more efficient stalling of RNA-polymerase, which could aggravate downstream pathological consequences. Indeed, mice models of ALS show accelerated disease progression when intracellular environment is slightly acidified (61). Small changes in local pH influence whether AQU or NAN would be formed during transcription of *C9orf72*. Based on described structural differences we can expect that AQU and NAN could bind and recruit different proteins. It was shown, for example, that replication protein A, a ssDNA binding protein known to unfold G-quadruplexes (62), binds to d(G_4_C_2_)_8_*in vitro* with significantly higher affinity at pH 5.0 compared to 7.8, suggesting it recognizes a structure which is preferred at acidic conditions (63). A cell could respond to acidification by using different molecular pathways triggered by binding to AQU compared to NAN.

5-Methyl-cytosine (5Me-dC) is an important epigenetic modification of cytosines found in CpG islands, which can alter expression of associated genes. In *C9orf72* linked ALS and FTD, hypermethylation of the CpG island located 5′ of the expanded d(G_4_C_2_)_*n*_•(G_2_C_4_)n repeat is protective against neurodegeneration and is associated with longer disease duration and later age of disease onset (64–66). Additionally, it was shown that d(G_4_C_2_)-repeat itself is hypermethylated in patients which carry large d(G_4_C_2_) expansions (>90 repeats) (67). Cells in which expanded d(G_4_C_2_) repeats are methylated exhibit decreased production of toxic dipeptide-repeat proteins and fewer RNA foci, which suggests that repeat methylation diminishes disease severity (68). In the course of our study, we observed that methylation does not significantly affect the ability of oligonucleotides to adopt a G-quadruplex structures. Oligonucleotides in which dC residues are substituted with 5Me-dC readily adopt both AQU and NAN, and the ratio at which the two structures form is not affected by methylation. However, since cytosines are on the surface of AQU and NAN (in the loops), methylation could affect which proteins bind to methylated versus unmethylated G-quadruplexes. One study demonstrated that methylation influences *in vitro* interaction between the C-rich d(G_2_C_4_)_8_ oligonucleotide and heterogeneous nuclear ribonucleoprotein complex protein K (hnRNP K) (69), a protein involved in chromatin remodeling, transcription, splicing and translation (70). Perhaps, G-quadruplex–protein interactions which have pathological consequences are weakened by the presence of 5Me-dC modification, which can explain why repeat-methylation appears to act protective against neurodegeneration (68). Alternatively, some structures which are formed at lower populations *in vitro* could become favored upon methylation of d(G_4_C_2_) repeats in the cell, which would effectively lower the concentration of G-quadruplexes and diminish the level of pathological molecular events they trigger (8).

## DATA AVAILABILITY

Atomic coordinates and list of chemical shifts have been deposited with following accession numbers: PDB ID 5OPH, BMRB ID 34168.

## Supplementary Material

Supplementary Data
