# Supplementary material for: NMR structure of a G-quadruplex formed by four d(G4C2) repeats: insights into structural polymorphism
Source: Nucleic Acids Res. 2018 Oct 2;46(21):11605–17. doi: 10.1093/nar/gky886 (PMC6265483; doi:10.1093/nar/gky886)
Supplement: Supplementary Data [file gky886_supplemental_files.pdf]

Supplementary Data

NMR structure of a G-quadruplex formed by four d(G_4C_2) repeats: insights into structural polymorphism

Jasna Brčić[1] and Janez Plavec[1,2,3,*]

[1] Slovenian NMR Center, National Institute of Chemistry, Ljubljana, SI-1000, Slovenia
[2] Faculty of Chemistry and Chemical Technology, University of Ljubljana, Ljubljana, SI-1000, Slovenia
[3] EN-FIST Center of Excellence, Ljubljana, SI-1000, Slovenia

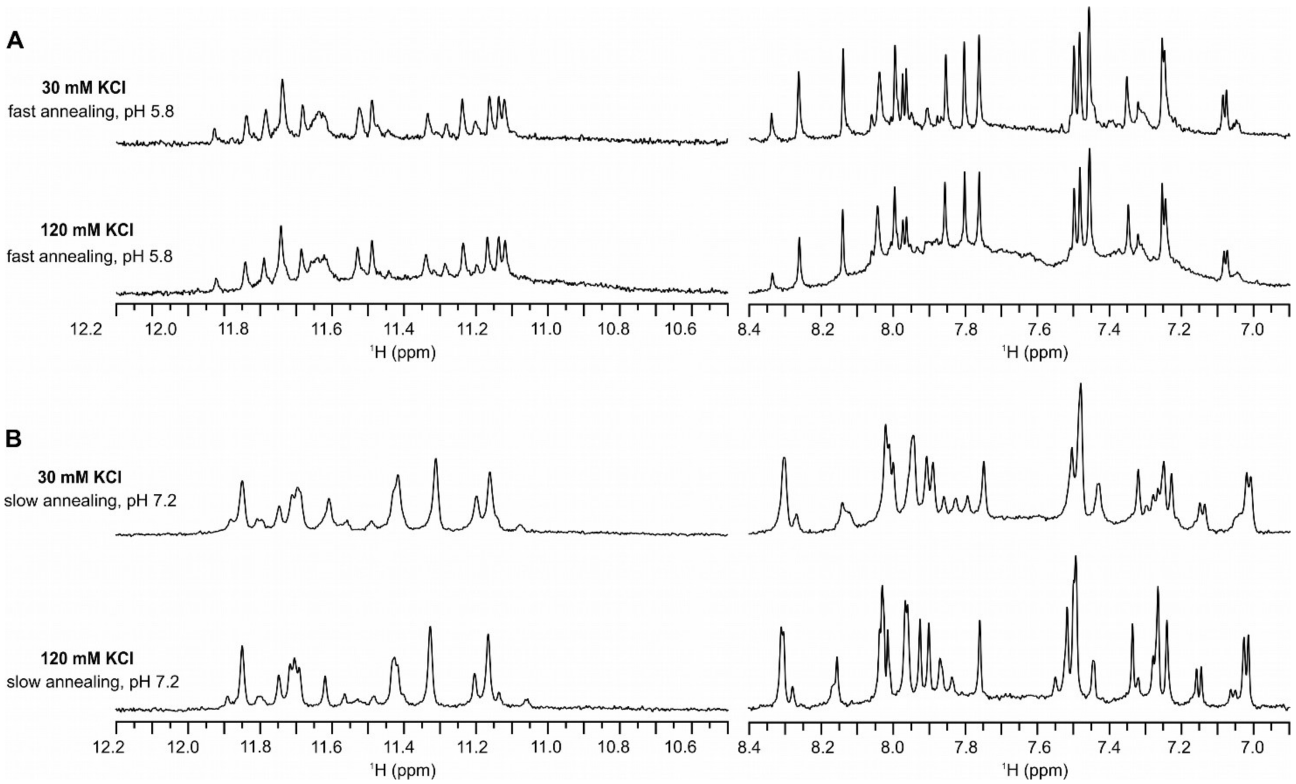

Figure S1. Imino and aromatic regions of the ^{1}H NMR spectra of sl21 in the presence of K^+ ions. Folding conditions are indicated next to individual spectrum. A) Fast annealing in the presence of 30 or 120 mM KCl and pH 5.8. B) Slow annealing in the presence of 30 or 120 mM KCl and pH 7.2. Spectra were recorded at 800 (A) or 600 MHz (B), 25 °C in 10% ^2H_2O, 90% H_2O, 30 or 120 mM KCl, pH 5.8 or 7.2 (20 mM K-phosphate buffer) and oligonucleotide concentrations around 0.1 mM.

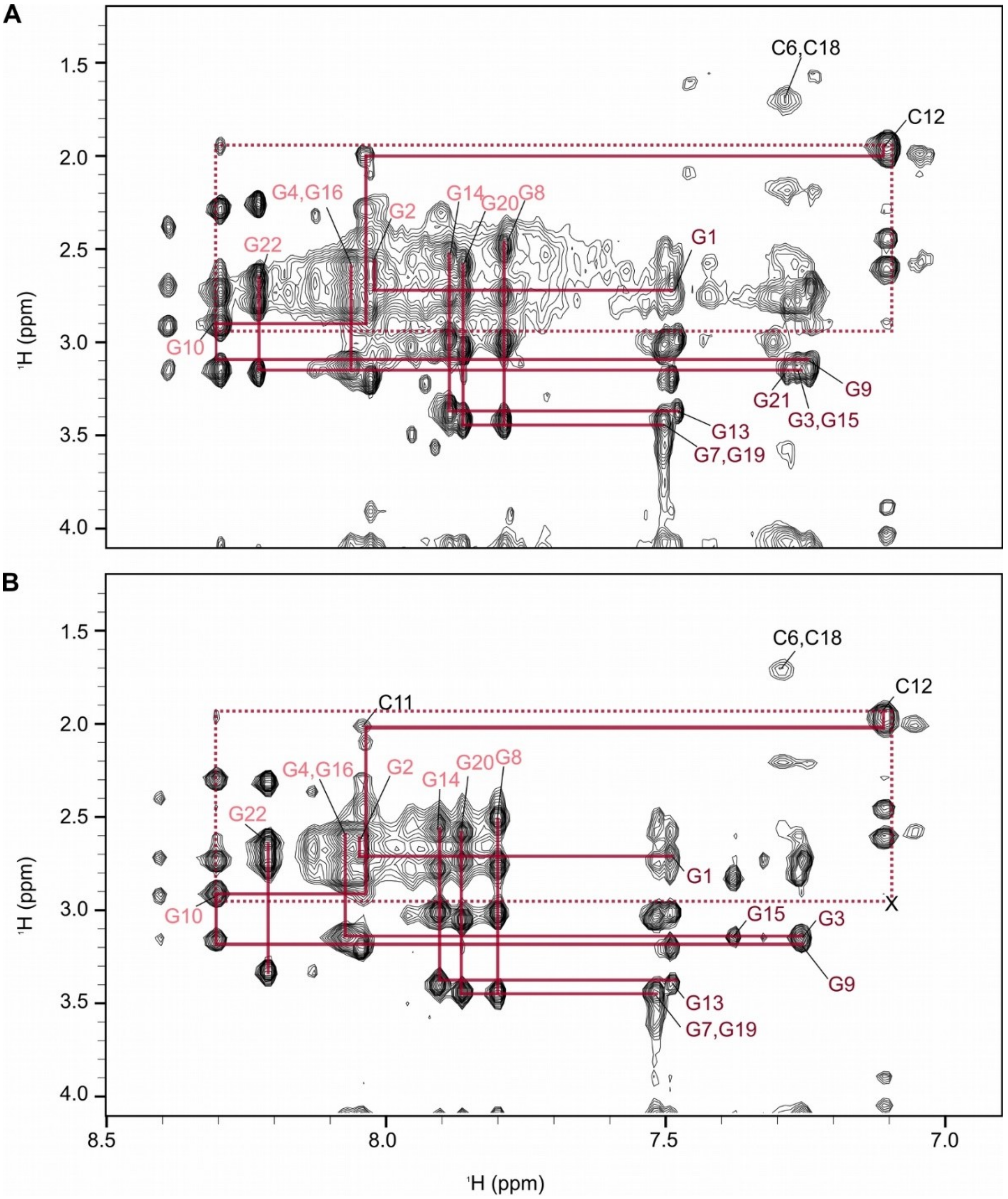

Figure S2. The H6/H8-H2′/H2″ region of NOESY (τ_m=200 ms) spectra and sequential walk for AQU adopted by wt22 (A) and sl21 (B). Assignments are shown next to the intraresidual H6/H8(n)-H2’(n) cross-peaks. The lines that connect the aromatic H6/H8-H2’ cross-peaks are depicted with solid (between sequential residues) and dotted red lines (between non-sequential residues). Assignments corresponding to *syn* guanines are shown in pink, *anti* guanines in red and cytosine residues in black. G10H2’-C12H6 cross-peak visible at higher vertical scale is marked with a letter X. NOESY spectra were recorded at 600 MHz, 5 °C in 100% ^2H_2O, 30 mM KCl, pH 5.8 (20 mM K-phosphate buffer) and oligonucleotide concentration of 0.4 mM.

Table S1 NMR chemical shifts of AQU adopted by sl21, determined at 25 °C.

Residue	H1	NH2	H6/H8	H5	H1’	H2’	H2”	H3’	H4’	H5’/H5”
G1	11.68	na	7.45		6.10	2.74	3.14	4.96	4.37	na
G2	11.73	na	8.00		6.17	2.60	2.79	5.07	4.47	na
G3	11.12	8.82/6.30	7.25		5.97	3.14	2.77	5.06	4.52	na
G4	11.64	9.90/6.35	8.03		6.00	2.58	2.70	4.97	4.44	na
C5		na	7.40	na	5.34	1.71	2.21	4.54	4.11	3.90/4.20
C6		na	7.30	5.31	5.47	1.72	2.48	4.55	na	3.50/3.54
G7	11.65	na	7.48		6.06	3.44	2.99	4.74	4.43	na
G8	11.73	na	7.25		6.20	2.48	2.74	4.98	4.51	na
G9	11.13	8.68/6.41	8.26		5.91	3.14	2.70	5.07	4.50	na
G10	11.52	na	7.46		5.93	2.89	2.33	5.07	4.42	na
C11		na	7.97	6.01	5.91	1.92	2.38	4.74	4.25	na
C12		na	7.08	4.51	5.91	1.93	2.54	4.75	4.19	na
G13	11.16	9.39/6.79	7.46		6.10	3.40	2.98	4.87	4.42	na
G14	11.32	na/6.76	7.85		6.17	2.52	2.74	5.00	4.46	na
G15	11.24	8.81/6.28	7.36		5.97	3.14	2.77	5.06	4.53	na
G16	11.64	9.90/6.35	8.03		6.00	2.58	2.70	4.97	4.44	na
C17		na	7.40	na	5.34	1.71	2.21	4.54	4.35	3.90/4.20
C18		na	7.30	5.31	5.47	1.71	2.48	4.55	na	3.50/3.54
G19	11.65	na	7.51		6.06	3.45	3.00	4.74	4.43	na
G20	11.78	na	7.80		6.18	2.54	2.73	4.99	4.51	na
^{Br}G21	11.49	8.84/6.51			6.10	3.34	2.71	5.09	4.46	na
G22	11.81	8.85/6.51	8.14		6.11	2.60	2.31	4.76	4.19	na

* Protons that have not been assigned are marked with na (non-assigned).

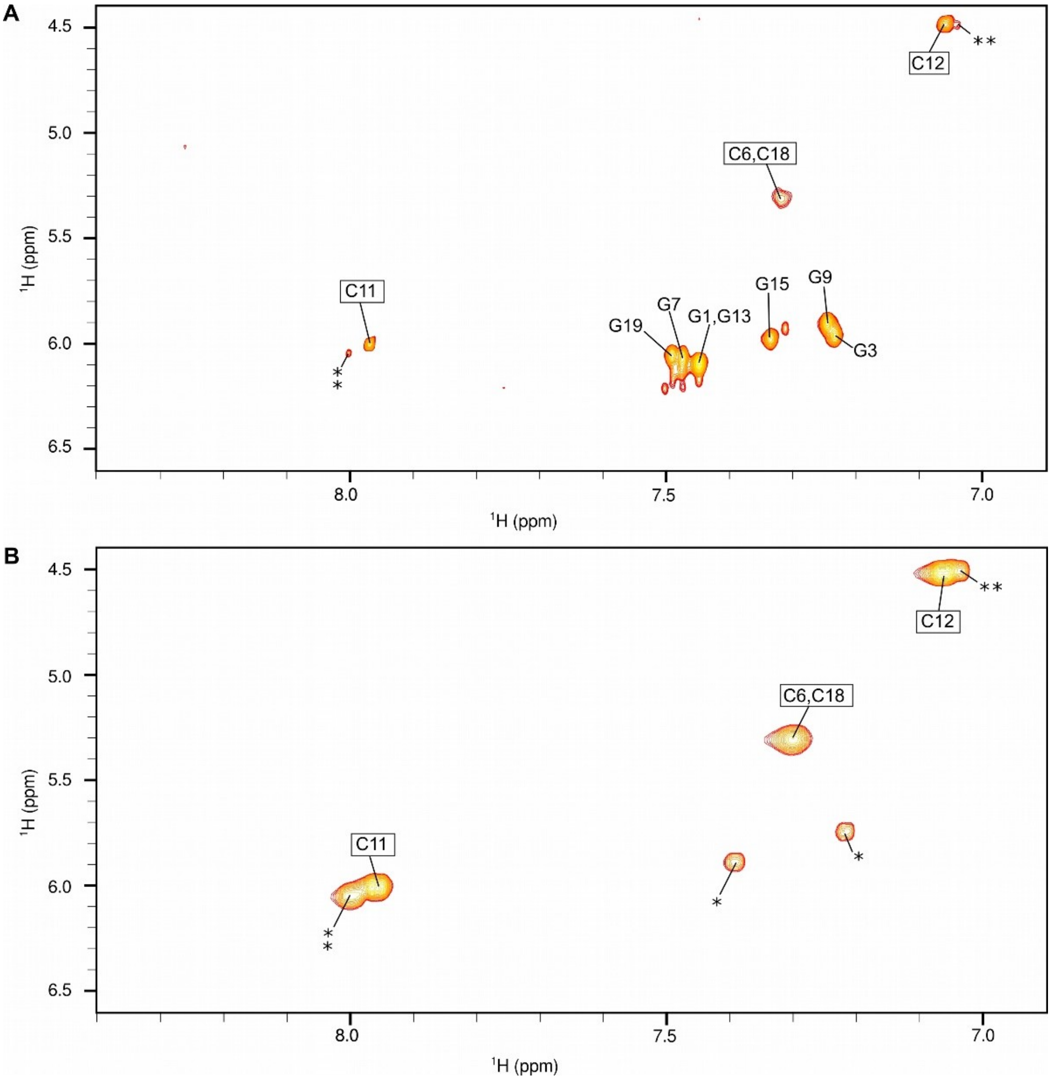

Figure S3. Assignment of guanine residues in *syn* glycosidic conformation and H5-H6 cross-peaks of cytosine residues in AQU adopted by sl21. Aromatic-sugar region of A) NOESY (τ_m=80 ms) and B) TOCSY (τ_m=60 ms) spectra of sl21. Assignments of guanine residues in *syn* conformation are shown next to intraresidual H8-H1' cross-peaks. Assignments marked with rectangles denote H5-H6 cross-peaks of cytosine residues in AQU. The H5-H6 cross-peaks that correspond to cytosine residues of NAN, which is present as a minor species, are indicated by stars. Spectra were recorded at 800 (A) and 600 MHz (B), 25 °C in 100% ^2H_2O, 30 mM KCl, pH 5.8 (20 mM K-phosphate buffer) and oligonucleotide concentration of 0.4 mM.

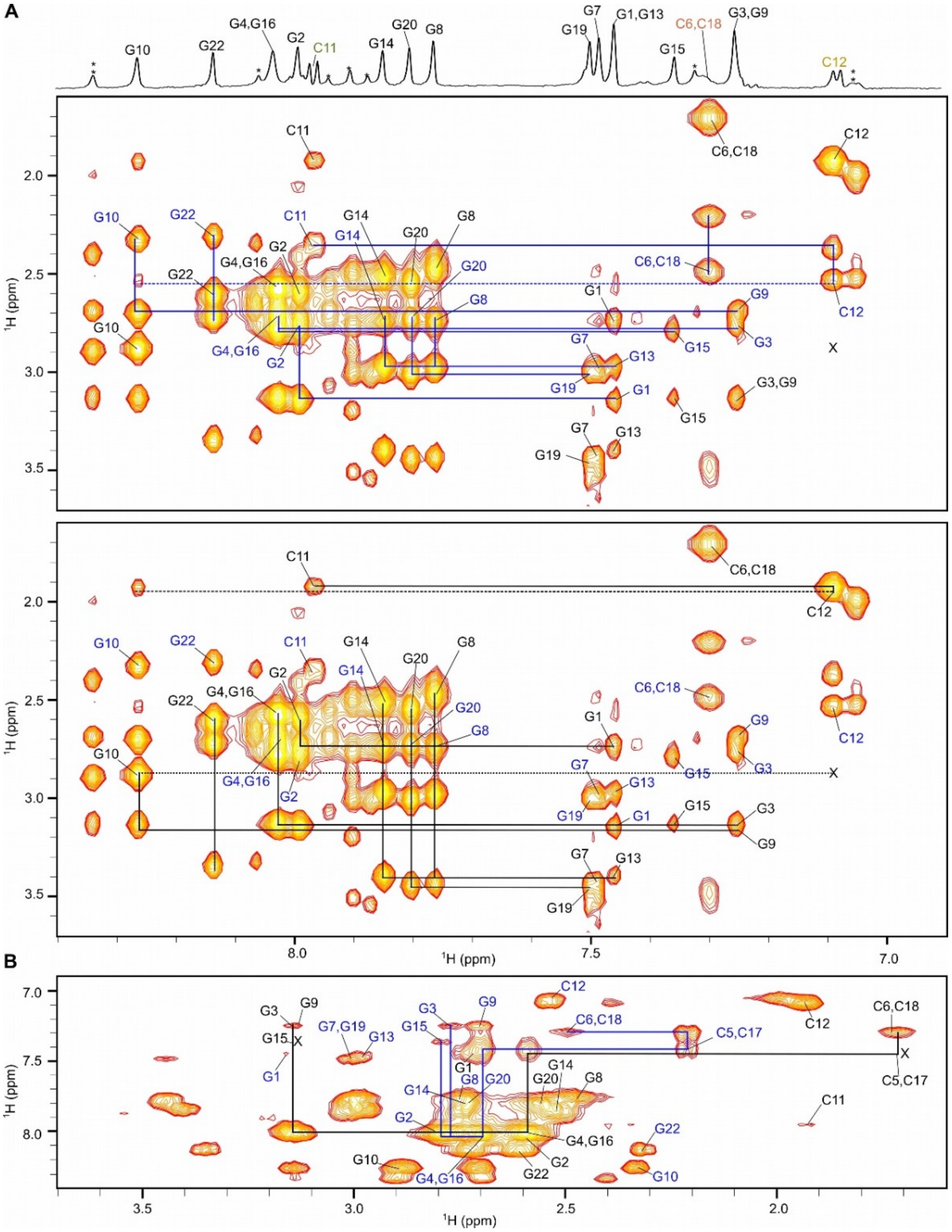

Figure S4. The H6/H8-H2'/H2" (A) and H2'/H2"-H6/H8 regions (B) of NOESY (τ_m=300 ms) spectrum. Assignments are shown next to the intraresidual H6/H8(n)-H2"(n) (blue) and H6/H8(n)-H2'(n) (black) cross-peaks. The lines that connect the H6/H8-H2"/H2' cross-peaks between sequential and non-sequential residues are depicted with solid and dotted lines, respectively. Sequential walk for the G3-C6 and G15-C18 segments is shown in panel B. Cross-peaks that are visible at a higher vertical scale are marked with a letter X. The unassigned cross-peaks at the C6H6 and C18H6 frequency line (7.30 ppm) in panel A correspond to the intranucleotide C6H5'/H5"-H6 and C18H5'/H5"-H6 contacts. NOESY spectrum was recorded at 600 MHz, 25 °C in 10% ^2H_2O, 90% H_2O, 30 mM KCl, pH 5.8 (20 mM K-phosphate buffer) and oligonucleotide concentration of 1.0 mM.

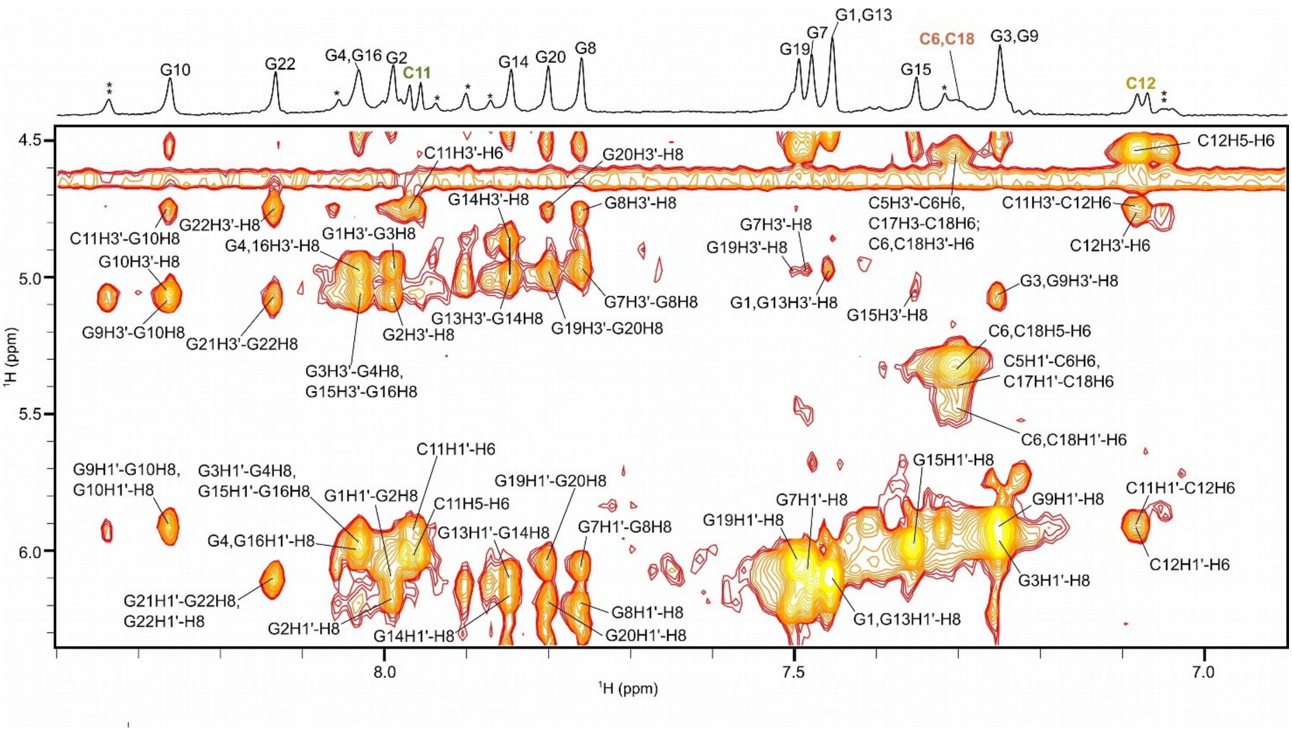

Figure S5. The H6/H8-H1′/H3’ region of NOESY (τ_m=250 ms) spectrum of AQU adopted by sl21 recorded at 25 °C. Assignments are shown next to the cross-peaks. The 1D ^{1}H NMR spectrum of sl21 is shown above the 2D plot with assignment of H6 and H8 signals corresponding to AQU. Signals of NAN, which is present as a minor species, are indicated with stars. NOESY spectrum was recorded at 600 MHz, 25 °C in 10% ^2H_2O, 90% H_2O, 30 mM KCl, pH 5.8 (20 mM K-phosphate buffer) and oligonucleotide concentration of 0.4 mM.

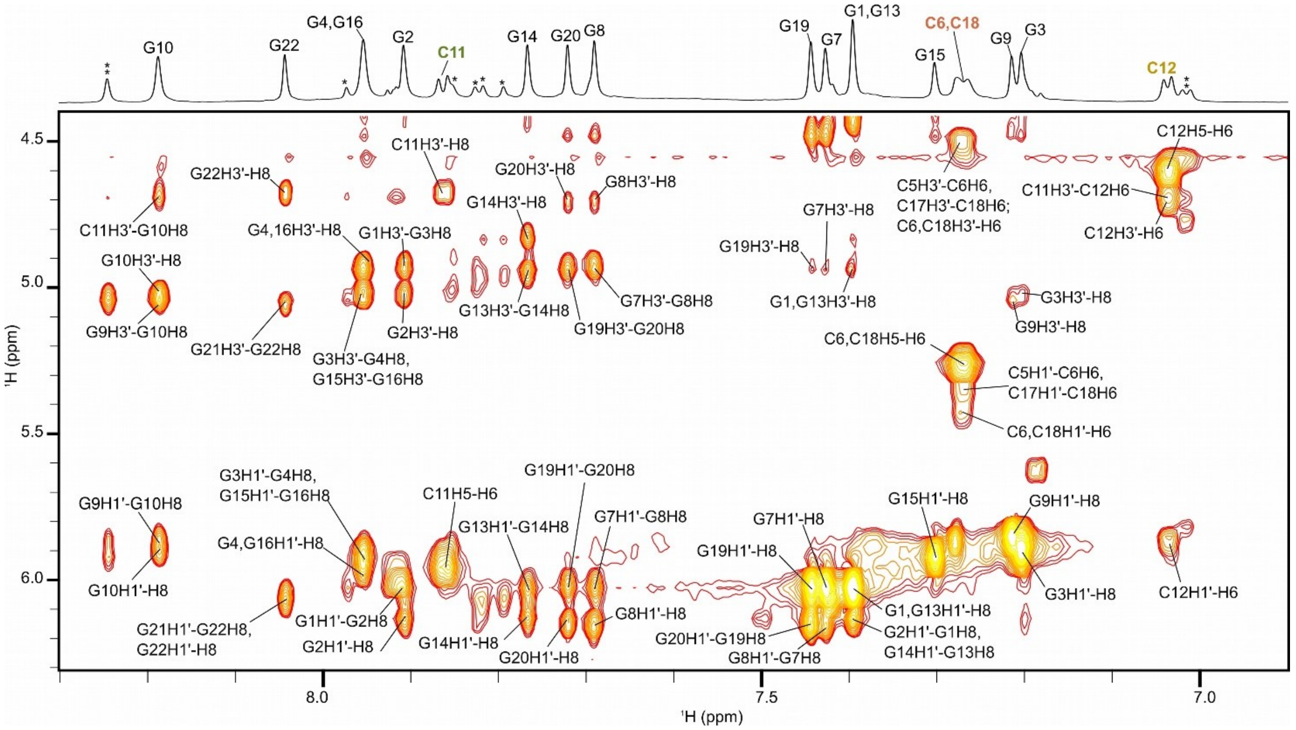

Figure S6. The H6/H8-H1′/H3’ region of NOESY (τ_m=300 ms) spectrum of AQU adopted by sl21 recorded at 45 °C. Assignments are shown next to the cross-peaks. The 1D ^{1}H NMR spectrum of sl21 is shown above the 2D plot with assignment of H6 and H8 signals corresponding to AQU. Signals of NAN, which is present as a minor species, are indicated with stars. NOESY spectrum was recorded at 800 MHz, 45 °C in 10% $^{2}H_2O$, 90% H_2O, 30 mM KCl, pH 5.8 (20 mM K-phosphate buffer) and oligonucleotide concentration of 1.0 mM.

Assignment of C5, C6, C17 and C18 with the help of 5Me-dC substitutions

Symmetry, spectral overlap and signal broadening made the assignment of C5, C6, C17 and C18 difficult. However, most of the signals corresponding to C5, C6, C17 and C18 protons could be assigned (Table S1). The following signals were not visible in any of the recorded spectra of sl21: i) amino NH2 protons of C5, C6, C17 and C18 ii) aromatic H5 proton of C5 and C17 iii) sugar H4' of C6 and C18. The assignment of C5, C6, C17 and C18 was confirmed by comparison between the NOESY spectra of sl21 and oligonucleotides with dC to 5Me-dC substitutions (Table S2).

Table S2: Sequences of oligonucleotides with 5Me-dC substitutions.

**Oligonucleotide name**	**Sequence (5'→ 3')**						
sl21[C5]	d[GGGG	^{Me}CC	GGGG	CC	GGGG	CC	GG^{Br}GG]
sl21[C6]	d[GGGG	C^{Me}C	GGGG	CC	GGGG	CC	GG^{Br}GG]
sl21[C17]	d[GGGG	CC	GGGG	CC	GGGG	^{Me}CC	GG^{Br}GG]
sl21[C18]	d[GGGG	CC	GGGG	CC	GGGG	C^{Me}C	GG^{Br}GG]
sl21[C5, C17]	d[GGGG	^{Me}CC	GGGG	CC	GGGG	^{Me}CC	GG^{Br}GG]
sl21[C5, C18]	d[GGGG	^{Me}CC	GGGG	CC	GGGG	C^{Me}C	GG^{Br}GG]
sl21[C5, C6]	d[GGGG	^{Me}C^{Me}C	GGGG	CC	GGGG	CC	GG^{Br}GG]
sl21[C6, C18]	d[GGGG	C^{Me}C	GGGG	CC	GGGG	C^{Me}C	GG^{Br}GG]
sl21[C6, C17]	d[GGGG	C^{Me}C	GGGG	CC	GGGG	^{Me}CC	GG^{Br}GG]
sl21[C17, C18]	d[GGGG	CC	GGGG	CC	GGGG	^{Me}C^{Me}C	GG^{Br}GG]
sl21[C5, C6, C17, C18]	d[GGGG	^{Me}C^{Me}C	GGGG	CC	GGGG	^{Me}C^{Me}C	GG^{Br}GG]

*Modified residue 5Me-dC is represented as ^{Me}C. ^{Br}G represents G21 which is substituted with 8Br-dG in all of the sequences.

In 5Me-dC, the H5 on the base moiety is replaced by a methyl group. Consequently, the H5-H6 cross-peak in NOESY spectrum is not visible when dC is replaced by 5Me-dC in the sequence. NOESY spectra of oligonucleotides with dC to 5Me-dC substitutions show that one of the H5-H6 cross-peaks disappears only when C6 and C18 are simultaneously replaced by 5Me-dC (Figure S7). This indicates that a single H5-H6 cross-peak corresponds to overlapped cross-peaks of C6 and C18. The H5-H6 cross-peak of C5 and C17 was not visible in the NOESY and TOCSY spectra of sl21. In addition, the H5-H6 cross-peak of C5 is missing in the NOESY spectra of the methylated analogues of sl21, in which the C5H5-H6 would be expected (sl21[C6], sl21[C18], sl21[C6, C18] and sl21[C17]). Similarly, the H5-H6 cross-peaks of C17 is not visible in the NOESY spectra of sl21[C6], sl21[C18], sl21[C6, C18] and sl21[C5] (Figure S7).

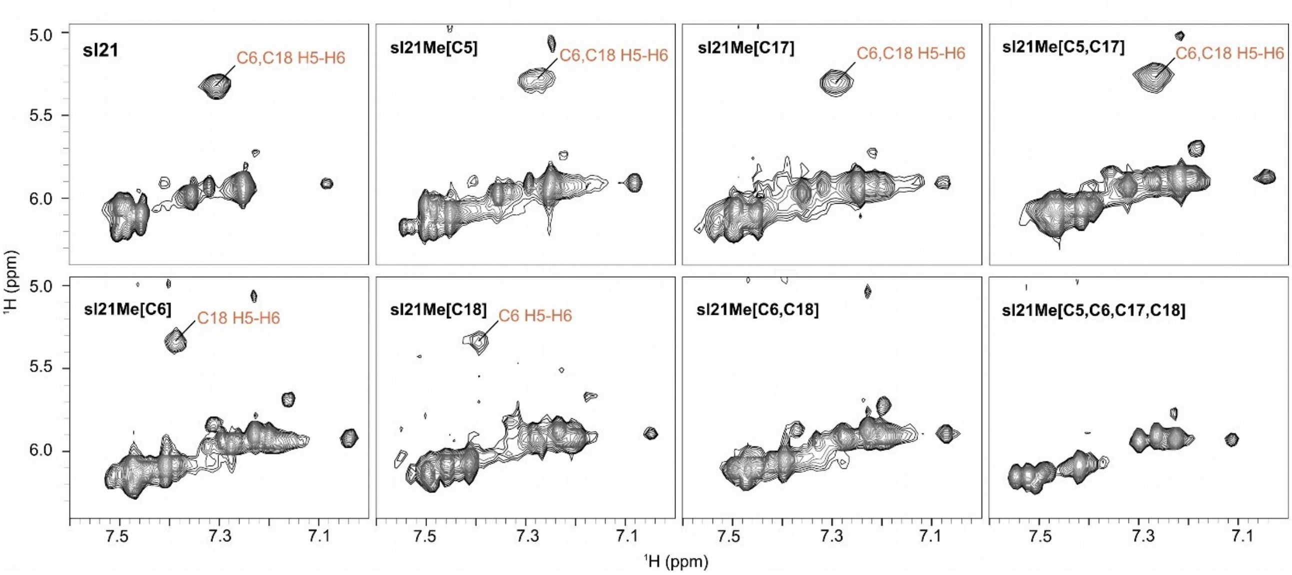

Figure S7. Assignment of the H5-H6 cross-peak of C6 and C18. Part of the aromatic-anomeric region of NOESY (τ_m=250 ms) spectra of sl21 and oligonucleotides with dC to 5Me-dC substitutions at different positions. Oligonucleotide names are written in the top left corner of each individual spectrum with corresponding sequences written in Table S2. NOESY spectra were recorded at 25 °C in 10% ^2H_2O, 90% H_2O, 30 mM KCl, pH 5.8 (20 mM K-phosphate buffer) and oligonucleotide concentration around 0.3 mM (5Me-dC substituted oligonucleotides) and 0.4 mM (sl21).

Assignment of the H6 signals of C5, C6, C17 and C18 was further corroborated by spectral similarity of double 5Me-dC substituted oligonucleotides. The NOESY spectra of sl21 analogues with dC to 5MedC substitutions are very similar to the NOESY spectrum of sl21, which suggests that the general fold of AQU remains very similar upon methylation (Figures S7, S8 and S9). In sl21, the expected C5H6-G4H2',H2", C17H6-G16H2',H2", C5H6-C5H2',H2", and C17H6-C17H2',H2" cross-peaks in the H6/H8-H2'/H2" region of NOESY (τ_m=300 ms) spectrum are not visible since they are broadened to the baseline (panel A of Figure S4). Similarly, spectral analysis of oligonucleotides with 5Me-dC substations was complex, since some of the expected cross-peaks were not visible. For example, the NOE cross-peaks on the H6 resonance line of C5, C17, ^{Me}C5 and ^{Me}C17 in the aromatic H6/H8-H2'/H2" region of NOESY (τ_m=250 ms) spectra of the 5Me-dC substituted oligonucleotides are missing (Figure S8). However, the expected H6(n)-H2'/H2"(n) cross-peaks of C6, C8, ^{Me}C6 and ^{Me}C18 are visible (Figure S8). By comparing the NOESY spectra of different methylated analogues of sl21, we could confirm the C2-axis of symmetry in the C5-C6 and C17-C18 loops and unequivocally show that C5 and C17, as well as C6 and C18 are isochronous. This is clearly demonstrated in Figure S8, where it can be seen that only certain combinations of double dC to 5Me-dC substitutions in the C5-C6 and C17-C18 loops break the apparent C2-symmetry. In sl21, there is a single set of overlapped NOE cross-peaks along the resonance line which corresponds to C6H6 and C18H6. Breaking of symmetry is expected to display as appearance of two distinct sets of cross peaks. Symmetry was retained when C6 and C18 were simultaneously replaced with 5Me-dC, which is seen as a single set of cross-peaks on the H6 resonance line of ^{Me}C6 and ^{Me}C18. Similarly, a single set of cross-peaks was observed for C6 and C18, when residues C5 and C17 were simultaneously substituted with 5Me-dC. Other combinations of double dC to 5Me-dC substitution result in breaking of the apparent C2-symmetry (Figure S8). For example, H6 signals of ^{me}C6 and C18 are resolved, which is shown as the appearance of two different sets of cross-peaks in the NOESY spectrum (τ_m=250 ms) of sl21[C6, C17] and sl21[C5, C6]. Likewise, two resolved sets of cross-peaks are observed on the resonance lines of H6 for ^{me}C18 and C6 in the NOESY spectrum (τ_m=250 ms) of sl21[C17, C18].

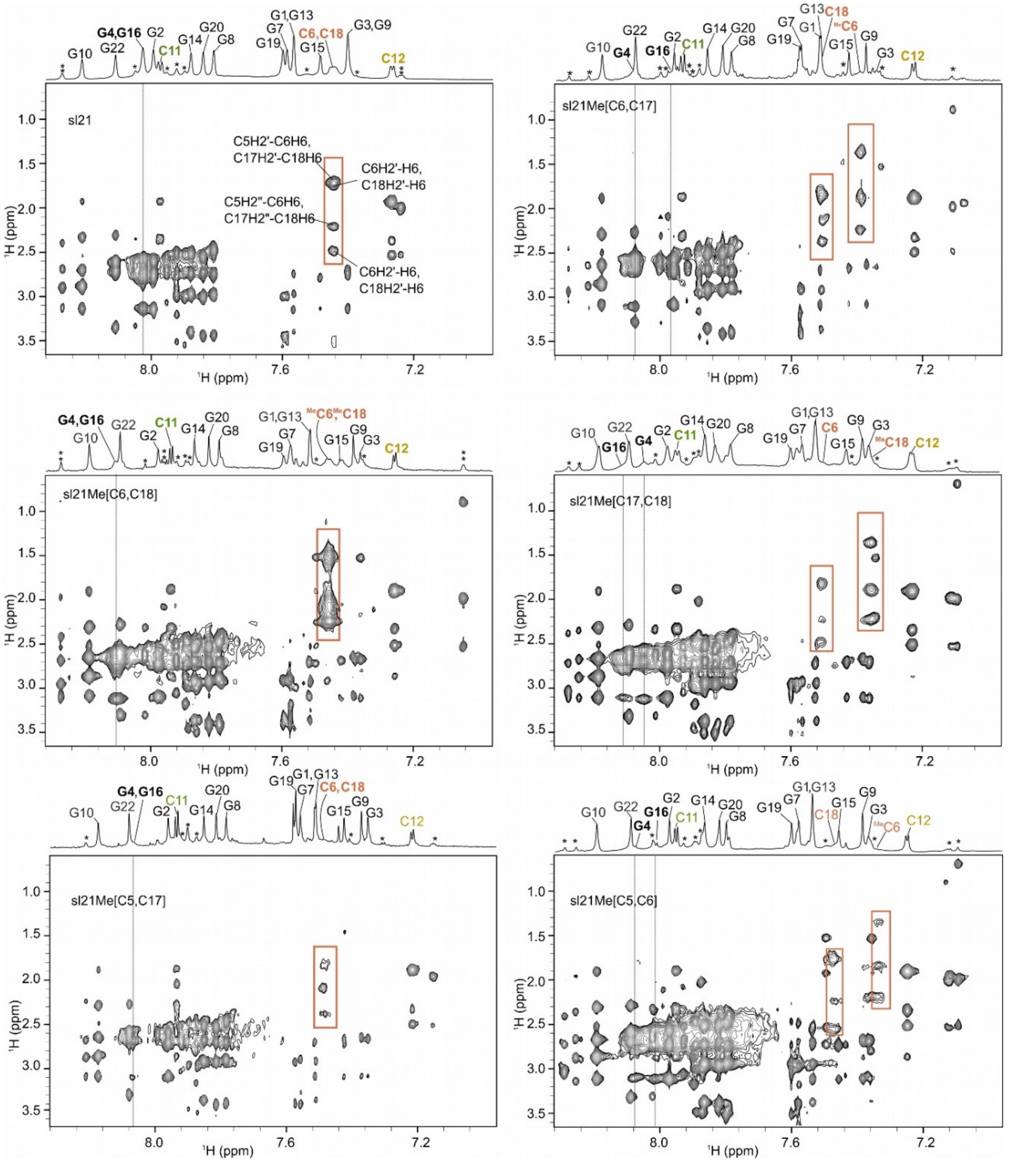

Figure S8. The H6/H8-H2'/H2" regions of NOESY (τ_m=250 ms) spectra of sl21 and oligonucleotides with different double dC to 5Me-dC substitutions. Oligonucleotide names are written in the top left corner of each individual spectrum with corresponding sequences written in Table S2. Orange rectangles are shown around the CH2',H2"(n-1)-CH6(n) and CH2',H2"(n)-H6(n) cross-peaks on the resonance line of H6 of C(n), which corresponds to C6, C18, ^{Me}C6 or ^{Me}C18. Vertical lines are shown along the resonance lines of H8 corresponding to G4 an G16 (in bold). The corresponding 1D ^{1}H NMR spectra with assignments of H6 and H8 signals of AQU are shown above the 2D plots. Signals corresponding to NAN, which is present as a minor species, are indicated by stars. NOESY spectra were recorded at 25 °C in 10% ^2H_2O, 90% H_2O, 30 mM KCl, pH 5.8 (20 mM K-phosphate buffer) and oligonucleotide concentrations around 0.3 mM (5Me-dC substituted oligonucleotides) and 0.4 mM (sl21).

In 5Me-dC, there is a methyl group instead of the H5 which is present at position 5 in residue dC. Oligonucleotides where residues C5, C6, C17 and C18 were replaced one-by-one or in pairs with 5Me-dC did not display methyl-H6 cross-peaks in their corresponding NOESY (τm=250 ms) spectra (Figure S8). The missing methyl-H6 cross-peaks of ^{Me}C5 and ^{Me}C17 in the NOESY spectra of sl21 analogues can be expected, since the analogous H5-H6 cross-peaks of C5 and C17 are not visible in the NOESY and TOCSY spectra of sl21 due to signal broadening. The missing methyl-H6 cross-peaks of ^{Me}C6 and ^{Me}C18 can also be attributed to signal broadening, as it was clearly demonstrated that C6 and C18 as well as C5 and C17 undergo conformational motions which are intermediate on the NMR chemical shift time-scale in AQU.

Dynamics of the C5-C6 and C17-C18 loops is possibly affected by the presence of the methyl groups on 5Me-dC residues. When C5, C6, C17 and C18 are simultaneously replaced by 5Me-dC in sl21[C5, C6, C17, C18], two weak methyl-H6 cross-peaks are visible in the NOESY (τ_m=400 ms) spectrum recorded at 25 °C (Figure S9). One of the methyl-H6 cross-peaks can be assigned to residues ^{Me}C6 and ^{Me}C18 and one to the residues ^{Me}C5 and ^{Me}C17. The methyl-H6 cross-peaks corresponding to NAN, which is present as a minor species at around 20% population, are also visible in the spectrum. In addition, several NOE cross-peaks involving the methyl protons (MeH) of ^{Me}C5, ^{Me}C6, ^{Me}C17 and ^{Me}C18 could be assigned, namely a) ^{Me}C5MeH-G4H8, ^{Me}C17MeH-G16H8, b) ^{Me}C6MeH-H6, ^{Me}C18MeH-H6, c) ^{Me}C5MeH-G4H2”, ^{Me}C17MeH-G4H2”, d) ^{Me}C6MeH-^{Me}C5H2”, ^{Me}C18MeH-^{Me}C17H2”, e) ^{Me}C5MeH-H6, ^{Me}C17MeH-H6 (Figure S9). If we consider that the methyl group in 5Me-dC is analogous to the H5 proton in dC, the observed NOE contacts involving the methyl protons in the NOESY spectrum of sl21[C5, C6, C17, C18] correlate well with the position of the loop residues C5, C6, C17 and C18 in the structure of AQU adopted by (unmethylated) sl21.

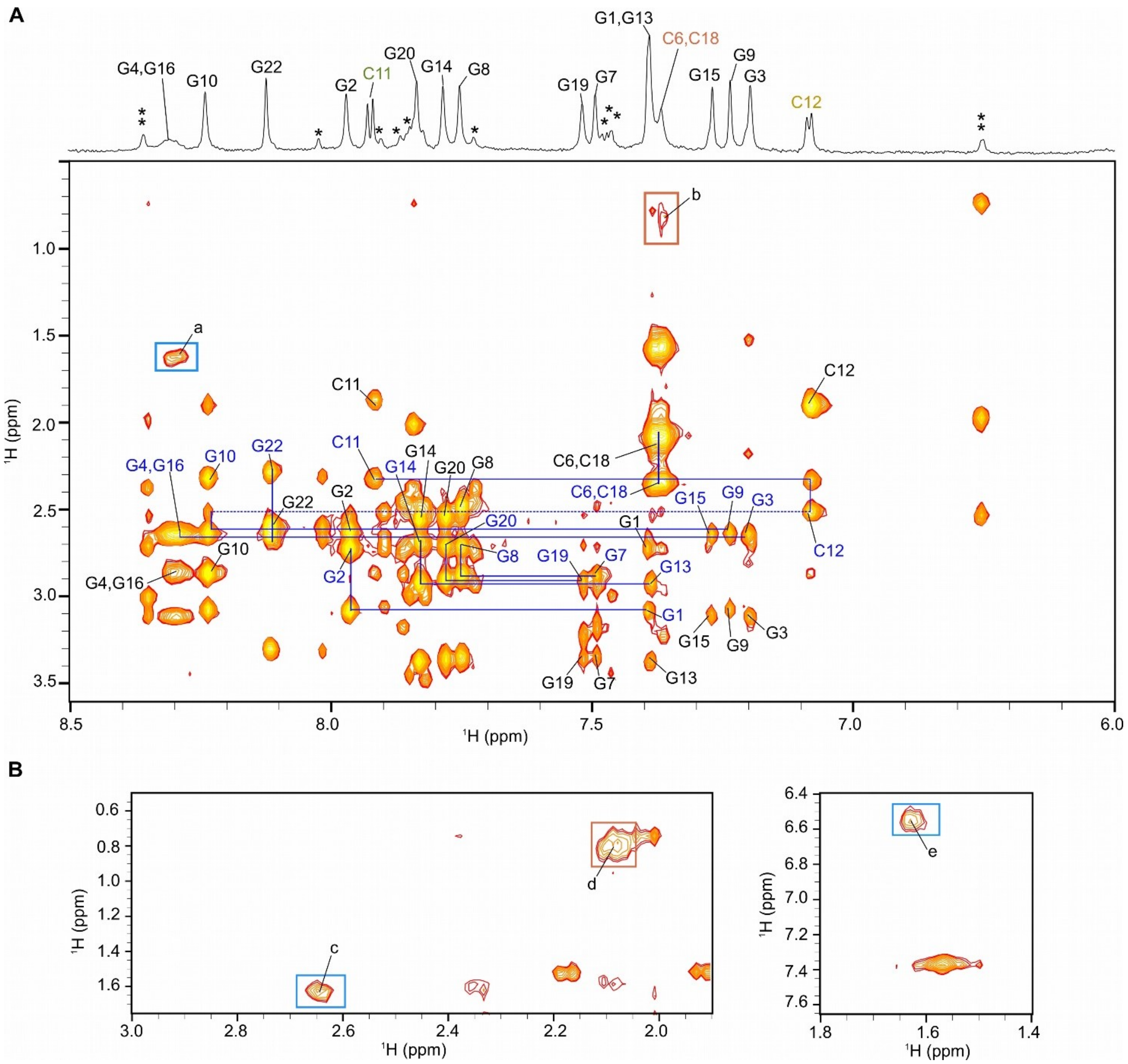

Figure S9. NOESY spectrum of AQU adopted by sl21[C5, C6, C17, C18]. A) The H6/H8-H2'/H2" region of NOESY (τ_m=400 ms) spectrum. Assignments are shown next to the intraresidual H6/H8(n)-H2"(n) (blue) and H6/H8(n)-H2'(n) (black) cross-peaks. The lines that connect the H6/H8-H2" cross-peaks between sequential and non-sequential residues are depicted with solid and dotted blue lines, respectively. B) The H2'/2"-methyl (left) and part of methyl-aromatic (right) regions of NOESY (τ_m=400 ms) spectrum. NOE cross-peaks involving the methyl protons (MeH) of 5Me-dC residues are highlighted with rectangles in blue (^{Me}C5 and ^{Me}C17) and orange (^{Me}C6 and ^{Me}C18). The labeled NOE cross-peaks correspond to a) ^{Me}C5MeH-G4H8, ^{Me}C17MeH-G16H8; b) ^{Me}C6MeH-H6, ^{Me}C18MeH-H6; c) ^{Me}C5MeH-G4H2", ^{Me}C17MeH-G16H2"; d) ^{Me}C6MeH-^{Me}C5H2", ^{Me}C18MeH-^{Me}C17H2"; e) ^{Me}C5MeH-H6, ^{Me}C17MeH-H6. The 1D ^{1}H NMR spectrum of sl21[C5, C6, C17, C18] is shown above the 2D plot with assignment of H6 and H8 signals corresponding to AQU. Signals corresponding to NAN, which is present as a minor species, are indicated by stars. All spectral regions are shown at the same vertical scale. NOESY spectrum was recorded at 800 MHz, 25 °C in 10% ^2H_2O, 90% H_2O, 30 mM KCl, pH 5.8 (20 mM K-phosphate buffer) and oligonucleotide concentration of 0.3 mM.

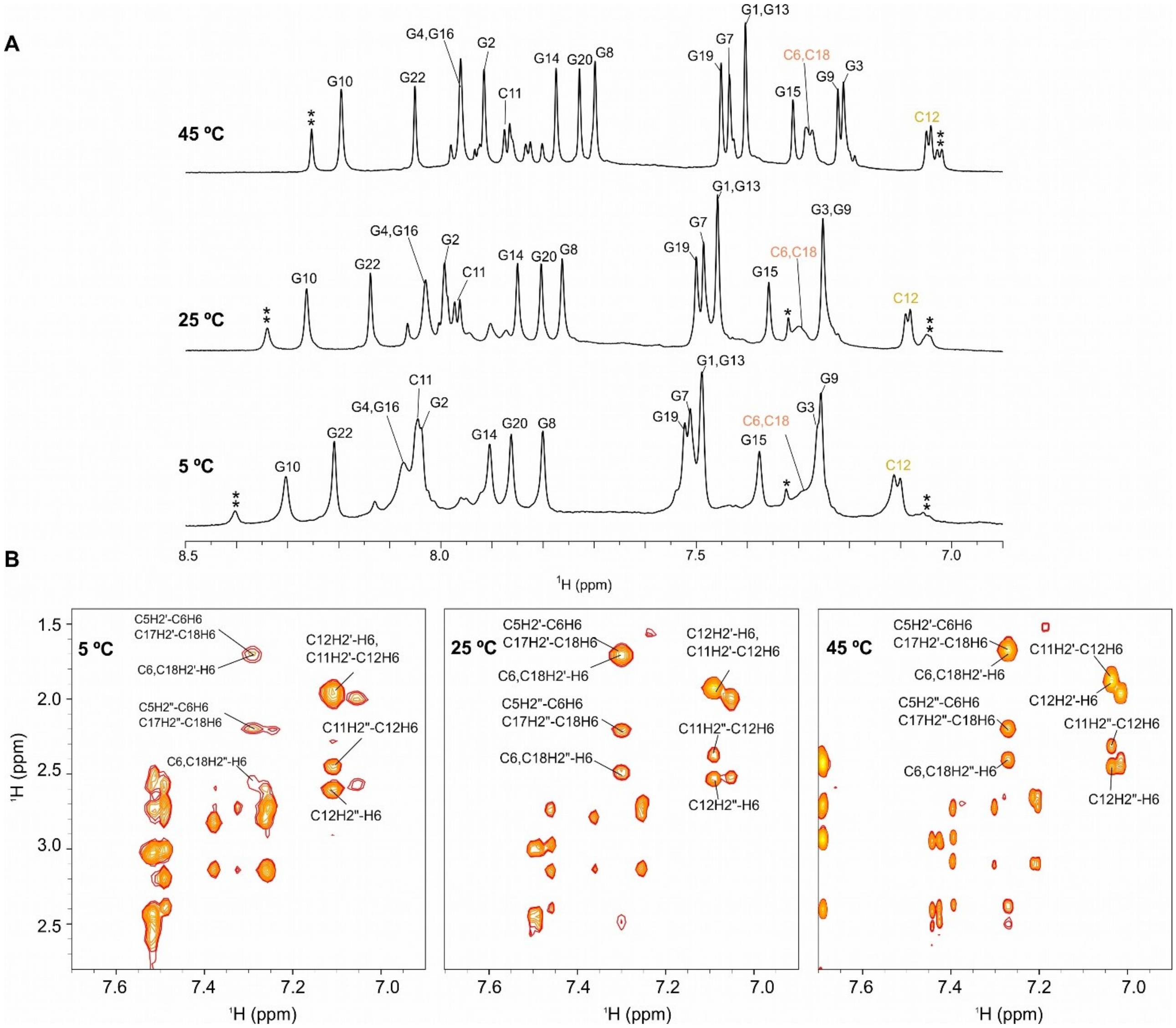

Figure S10: NMR spectra recorded at different temperatures reveal conformational dynamics of loop residues. A) Aromatic regions of 1D ^{1}H NMR spectra of AQU adopted by sl21 recorded at 5, 25 and 45 °C with corresponding assignment of H8 and H6 signals of AQU. Signals corresponding to NAN, which is present as a minor species, are indicated by stars. B) Part of sugar H6/H8-H2/H2” regions of NOESY (τ_m=300 ms) spectra recorded at different temperatures. NOESY spectra were recorded at 600 (at 5 and 25 °C) or 800 MHz (at 45 °C) in 10% $^{2}H_2O$, 90% H_2O, 30 mM KCl, pH 5.8 (20 mM K-phosphate buffer) and oligonucleotide concentration of 1.0 mM.

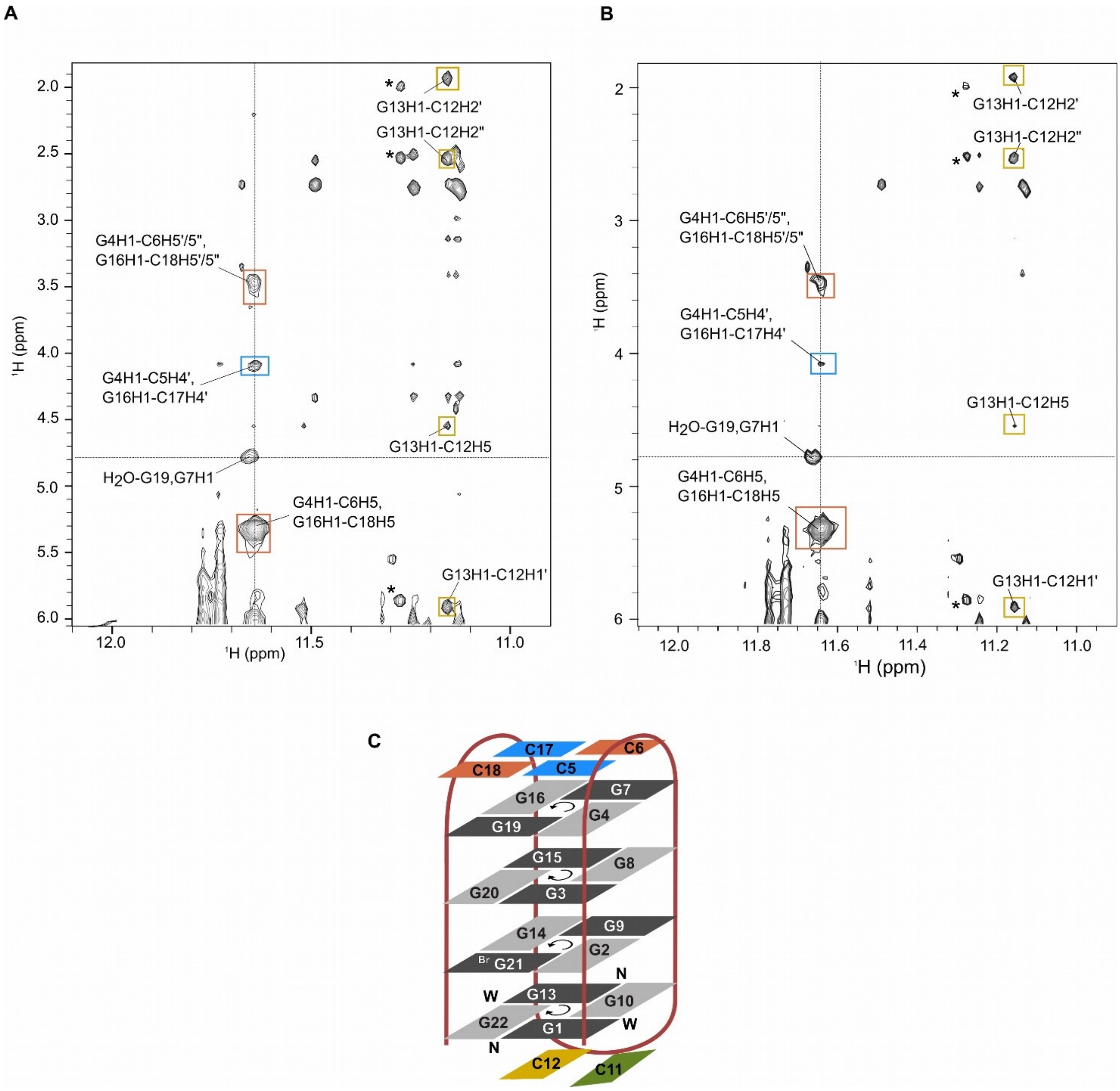

Figure S11: Imino-sugar region of NOESY spectra of AQU adopted by sl21 depicting structurally important cross-peaks between the loop residues and the nearby G-quartet. NOESY was recorded at A) 300 ms mixing time and B) 150 ms mixing time. NOE cross-peaks involving cytosine residues are highlighted with rectangles in blue (C5 and C17), orange (C6 and C18), and yellow (C12). Vertical line is shown along the resonance of G4H1 and G16H1. Horizontal line is shown along the resonance corresponding to bulk water. Cross-peaks can be observed between bulk water and H1 of G7 and/or G19. Cross-peaks of H1 and sugar protons between sequential guanine residues are also visible in this region. Stars mark the cross-peaks of NAN (C6H5,H2',H2"-G7H1 and C18H5,H2',H2"-G19H1), which is present as a minor species. NOESY spectra were recorded at 600 MHz, 25 °C in 10% $^{2}H_2O$, 90% H_2O, 30 mM KCl, pH 5.8 (20 mM K-phosphate buffer) and oligonucleotide concentration of 1.0 mM. C) The topology of AQU.

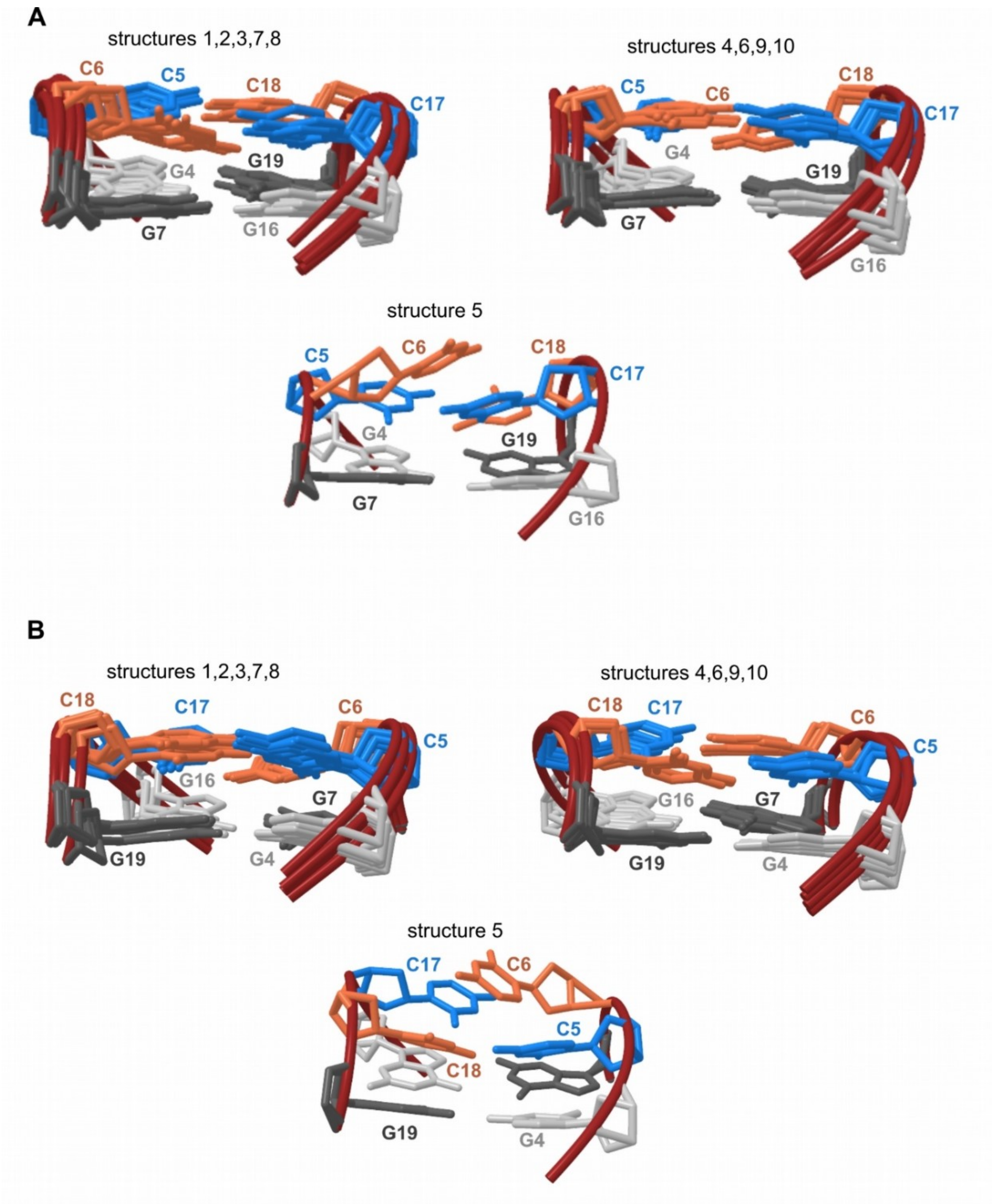

Figure S12. Structure of the lateral C5-C6 and C17-C18 loops in AQU in ten superimposed structures with the lowest energy and restraint violations. Individual structures of the ensemble are designated with a number. Side views of C5-C6 and C17-C18 loops stacked on the G4:G7:G16:G19 quartet in (A) and (B) are rotated with respect to one another by 180°. For clarity, cytosine residues are depicted in blue (C5 and C17), orange (C6 and C18), green (C11) and yellow (C12). Guanine residues in *syn* and *anti* glycosidic conformation are shown in dark and light gray color, respectively. Backbone atoms are shown as ribbon (red).

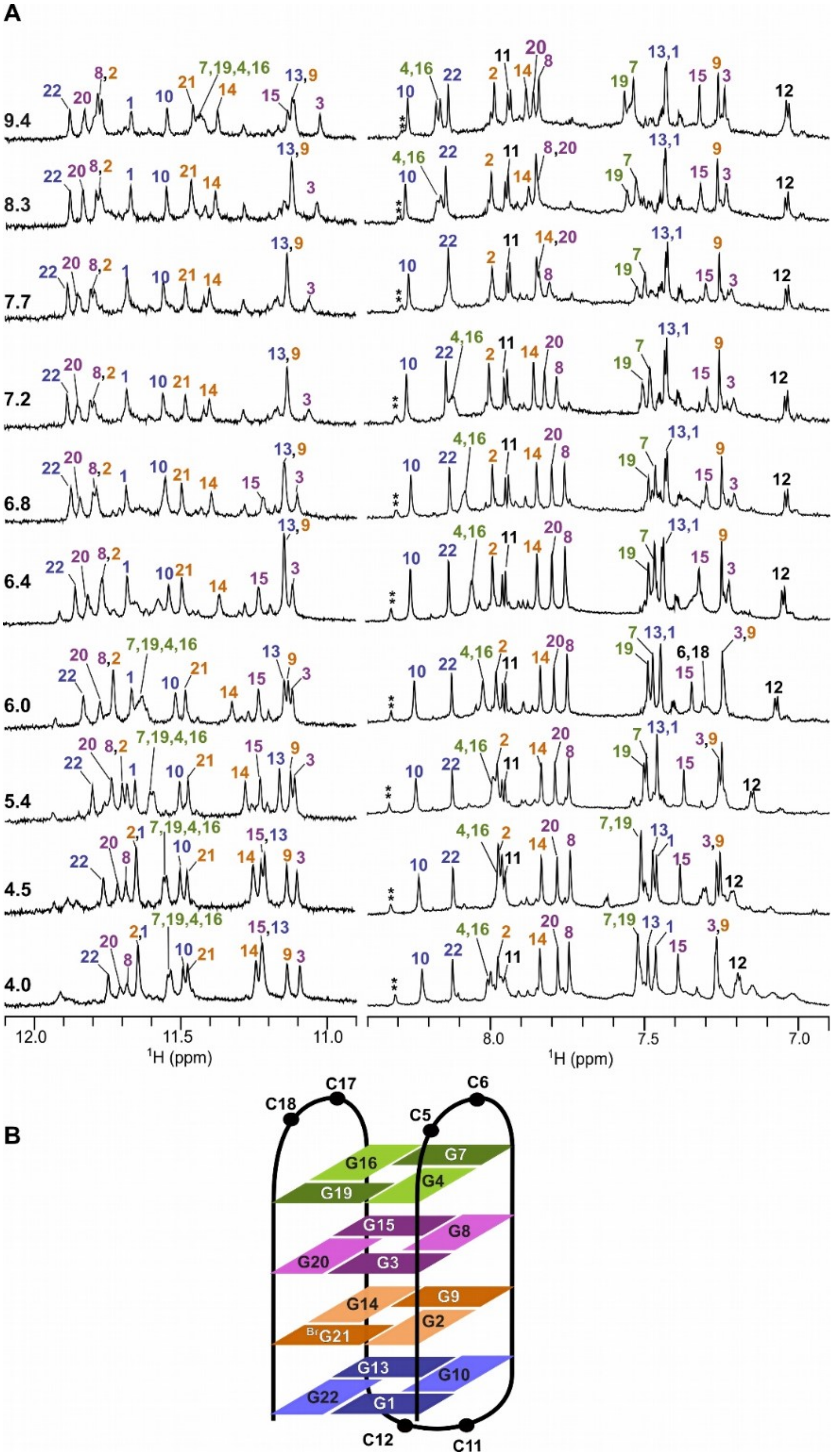

Figure S13. A) Variation of solution pH at room temperature after sl21 was folded into AQU (80%) and NAN (20%), by fast annealing at pH 5.8 (20 mM K-phosphate buffer) and 30 mM KCl. Stars indicate overlapped G4H8 and G16H8 signals of NAN, which is present as a minor species. B) Topology of AQU. Spectra were recorded at 800 MHz, 25 °C in 10% ^2H_2O, 90% H_2O, 30 mM KCl and oligonucleotide concentration of 0.1 mM.

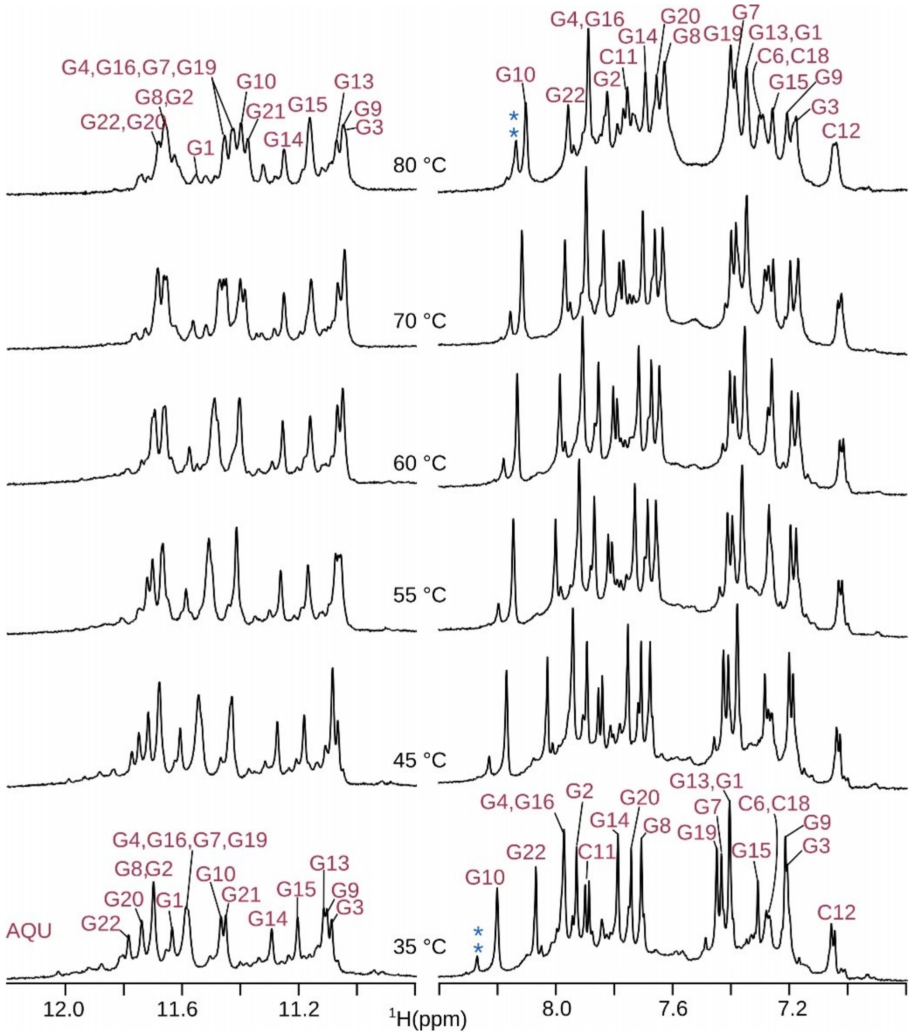

Figure S14. Imino and aromatic regions of variable temperature ^{1}H NMR spectra of sl21 with the assignment of signals corresponding to AQU shown in red at 35 °C and 80 °C. The spectra were recorded at 600 MHz in 10% ^2H_2O, 90% H_2O, 30 mM KCl, pH 5.8 (20 mM K-phosphate buffer) and oligonucleotide concentration of 0.1 mM. Resolved signals of NAN, which is present as a minor species, are indicated with stars in blue.

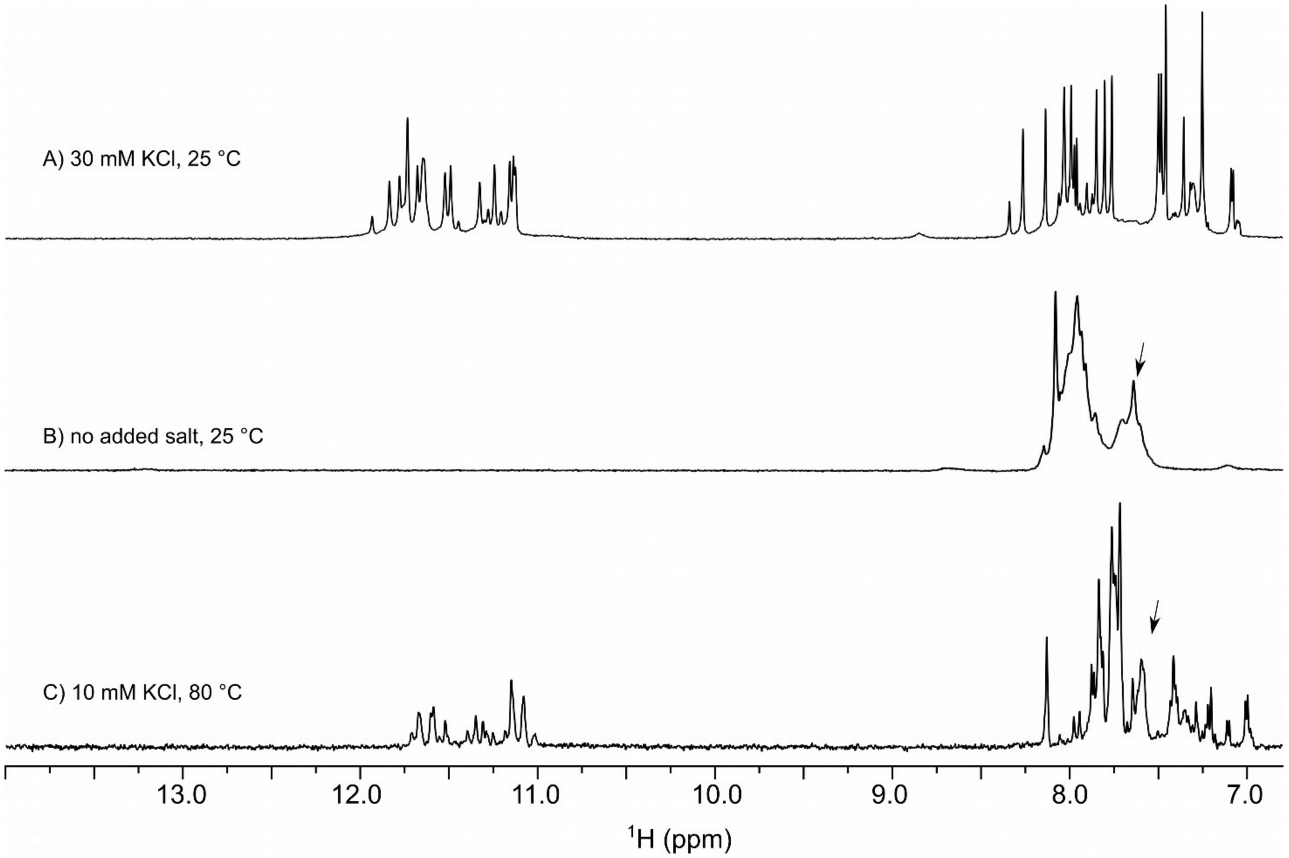

Figure S15. Imino and aromatic regions of ^{1}H NMR spectra of sl21 recorded at different solution and experimental conditions. A) At 25 °C, in the presence of 30 mM KCl and pH 5.8 (20 mM K-phosphate buffer), displaying spectral properties of sl21 folded into G-quadruplexes (80% AQU and 20% NAN). B) At 25 °C, recorded after purification and desalting steps with no added salts, displaying spectral properties of unfolded sl21. Solution pH has been adjusted to around 7 with LiOH. C) At 80 °C, in the presence of 10 mM KCl and pH 7.2 (20 mM K-phosphate buffer). Spectrum is the same as in Figure 6A. Arrow in panel C indicates the signals of aromatic protons, which are clearly distinguished from the signals of aromatic protons of AQU and NAN. Similar signal indicated by an arrow is present in the spectrum of unfolded sl21 in panel B. Spectra were recorded at 600 (A and C) or 800 MHz (B), 25 °C in 10% $^{2}H_2O$, 90% H_2O and oligonucleotide concentrations of 0.5 mM (A and B) or 0.1 mM (C).

ADDITIONAL COMMENTS TO EXPERIMENTAL METHODS

Sugar puckering

The H2/H2" proton resonances were stereospecifically assigned using DQF-COSY and NOESY (80 ms mixing time) spectra. Sugar puckering was assessed through analysis of DQF-COSY and TOCSY spectra and revealed that all guanine residues and C12 exhibit large $^3J_{H1'H2'}$ coupling constants, which is consistent with S-type sugar conformation. C11 displays a large $^3J_{H3'-H4'}$ coupling constant and $^3J_{H1'H2''}$ larger than $^3J_{H1'H2'}$, which is consistent with bias towards the N-type sugar conformation.

Folding of samples

Desalted samples were dried on a vacuum centrifuge, dissolved in H_2O and pH adjusted to approximately 6.5 with LiOH. Folding with slow annealing was achieved by heating the solution of DNA at 90 °C for 5 min prior to adding 120 or 30 mM KCl, 20 mM phosphate buffer with pH 7.2 and cooled slowly from 90 to 25 °C over the course of 16 h. Folding with fast annealing at pH 5.8 was achieved by heating the solution of DNA at 90 °C for 5 min prior to adding 30 mM KCl, 20 mM phosphate buffer with pH 5.8 and then immediately put on ice. Samples were left on ice for 2-8 hours, transferred to room temperature for 12-24 hours and then incubated for at least 24 hours at 40° C before experiments. To avoid aggregation, folding of all samples was done at DNA concentration in the range between 0.05 and 0.1 mM per strand. Completely folded samples were subsequently concentrated on Amicon Ultra Centrifugal Filters to reach their final concentration for NMR measurements. The sample for NMR studies in ^2H_2O was prepared by repeated lyophilization and final dissolution in 350 µl ^2H_2O. Samples for variable temperature NMR study were prepared at folding conditions that promote formation of AQU (fast annealing at pH 5.8) or NAN (slow annealing at pH 7.2). After samples were folded, the solvent was exchanged to aqueous solution containing 10 mM KCl and 20 mM K-phosphate with pH 7.2 for easier comparison between the spectra.

NMR restraints

Standard homonuclear 2D NMR experiments including 2D DQF-COSY, 2D TOCSY (20, 40, 60 and 80 ms mixing time) and NOESY (80, 150 and 250 ms mixing time) recorded at 25 °C in 100% ^2H_2O were used to assign non-exchangeable protons. Exchangeable proton resonances were assigned using 2D NOESY (60, 100, 150, 250, 300, 400 ms mixing time) recorded in 90% H_2O, 10% ^2H_2O at 5 and 25 °C. All NOE (Nuclear Overhauser Effect) distance restraints for non-exchangeable protons were obtained from 2D NOESY spectra recorded at 25 °C in 100% ^2H_2O with mixing times of 80 ms and 150 ms. The average volume of H5–H6 cross-peak of C11 was used as the distance reference of 2.45 Å. Cross-peaks were classified as strong (1.8–3.6 Å), medium (2.6–5.0 Å) and weak (3.5–6.5 Å). NOE distance restraints for exchangeable protons were obtained from 2D NOESY spectra recorded at 25 °C in 90% H_2O, 10% ^2H_2O with mixing times of 60, 150 and 300 ms. Cross-peaks of medium and weak intensity in 2D NOESY spectrum with a mixing time of 60 ms were classified as strong (1.8–3.6 Å) and medium (2.6–5.0 Å), respectively. Cross-peaks that were observed with medium intensity at 150 ms were also classified as medium (2.6–5.0 Å). Cross-peaks that appeared in 2D NOESY spectrum with a mixing time of 300 ms were classified as weak (3.5–6.5 Å). NOE contacts that involved protons of residues C5, C6, C17 and C18 were applied in calculations as distance restraints with looser upper

boundaries: (1.8–5.0 Å) for cross-peaks classified as strong and medium and (2.6–6.5 Å) for cross-peaks classified as weak. Torsion angle χ around the glycosidic bonds was restrained to a range between 25 and 95° for residues that were assigned a *syn* conformation and between 200 and 280° for residues that were assigned an *anti* conformation. Torsion angle χ around the glycosidic bond for residues C11 and C12 was restricted between 170 and 310°, the glycosidic torsion angles of C5, C6, C17 and C18 were left unrestrained. The pseudorotation phase angle (PPA) was used to restrict the sugar conformation into S-type with PPA between 162.0 and 180.0 and N-type with PPA values between 0.0 and 18.0. All guanine residues as well as C12 were restricted to S-type sugar conformation, while C11 was restricted to N-type. Sugar conformation for C5, C6, C17 and C18 was left unrestrained.

SA protocol

All calculations were initiated with random velocities. Generalized Born implicit model was used to account for solvent effects. The cut-off for non-bonded interactions was 20 Å and the SHAKE algorithm for hydrogen atoms was used with the tolerance of 0.0005 Å. In the first 50 ps of SA the temperature was raised from 300 to 1000 K. Molecules were held at constant temperature of 1000 K for 20 ps and then cooled to 300 K in the next 30 ps, after which the temperature was scaled down to 0 K in the last 30 ps. Restraints were included with following force constants: 40 kcal $mol^{-1}Å^{-2}$ for hydrogen bond restraints, 20 kcal mol^{-1} $Å^{-2}$ for NOE distances, 100 kcal mol^{-1} rad^{-2} for sugar pseudorotation phase angle restraints, 150 kcal mol^{-1} rad^{-2} for torsion angle χ and 20 kcal mol^{-1} $Å^{-2}$ for G-quartet base planarity restraints and 10 kcal mol^{-1} rad^{-2} for chirality restraints. Planarity restraints for G-quartets were excluded in the last 30 ps of SA. All 100 structures were minimized with a maximum of 20 000 steps of energy minimization. Planarity restraints were omitted in the minimization steps.
